# A hybrid mutational Northern Goshawk and elite opposition learning artificial rabbits optimizer for PEMFC parameter estimation

**DOI:** 10.1038/s41598-024-80073-2

**Published:** 2024-11-19

**Authors:** Pradeep Jangir, Absalom E. Ezugwu, Kashif Saleem, Arpita, Sunilkumar P. Agrawal, Sundaram B. Pandya, Anil Parmar, G. Gulothungan, Laith Abualigah

**Affiliations:** 1 University Centre for Research and Development, Chandigarh University, Gharuan, 140413 Mohali India; 2 Department of CSE, Graphic Era Hill University, Dehradun, 248002 India; 3 Department of CSE, Graphic Era Deemed To Be University, Dehradun, 248002 Uttarakhand India; 4 Applied Science Research Center, Applied Science Private University, Amman, 11931 Jordan; 5 Jadara University Research Center, Jadara University, Irbid, Jordan; 6 Unit for Data Science and Computing, North-West University, 11, Hofman Street, Potchefstroom, 2520 South Africa; 7 Department of Computer Science & Engineering, College of Applied Studies & Community Service, King Saud University, Riyadh, 11362 Saudi Arabia; 8 Department of Biosciences, Saveetha School of Engineering, Saveetha Institute of Medical and Technical Sciences, Chennai, 602 105 India; 9 Department of Electrical Engineering, Government Engineering College, Gandhinagar, 382028 Gujarat India; 10 Department of Electrical Engineering, Shri K.J. Polytechnic, Bharuch, 392 001 India; 11 Department of Electronics and Communication Engineering, SRM Institute of Science and Technology, SRM Nagar, Kattankulathur, Chengalpattu, 603203 Tamilnadu India; 12 Computer Science Department, Al al-Bayt University, Mafraq, 25113 Jordan; 13 Centre for Research Impact & Outcome, Chitkara University Institute of Engineering and Technology, Chitkara University, Rajpura, 140401, Punjab, India

**Keywords:** Proton Exchange membrane fuel cell parameter identification, Adaptive rabbits optimization, Mutation strategy, MNEARO, Optimization in Electrical Engineering, Chemical engineering, Electrical and electronic engineering, Fuel cells

## Abstract

For the purpose of simulating, controlling, evaluating, managing and optimizing PEMFCs it is necessary to develop accurate mathematical models. The present study develops a mathematical model which uses empirical or semi-empirical equations to estimate unknown model parameters through optimization techniques. This thesis calculates, analyzes and discusses the sum of squares error (SSE) between measured and estimated current and voltage values using parameters derived from multiple optimization techniques for six commercially available PEMFCs: BCS 500 W-PEMFC, 500 W SR-12 PEMFC, Nedstack PS6 PEMFC, H-12 PEMFC, HORIZON 500 W PEMFC and a 250 W-stack PEMFC. To minimize the SSE between measured and estimated current values under these new models we employ an advanced version of Artificial Rabbits Optimization called Mutational Northern goshawk and Elite opposition learning-based Artificial Rabbits Optimizer (MNEARO). Additionally SSE, Absolute Error (AE), and Mean Bias Error (MBE) are computed for different recent methods according to literature on voltage measurement. Other optimization algorithms including ARO, TLBO, DE and SSA are used for comparative analysis purposes. On top of that MNEARO outperforms others in terms of both computational cost as well as solution quality while experiments carried out using benchmark problems indicate its superiority over other meta-heuristics approaches.

## Introduction

Energy consumption has intensified and so has public consciousness on environmental issues, which have consequently led individual and government focus to alternative energy sources^1^. As a result, there is a growing need for further inquiry into various renewable energy alternatives such as solar power, wind and wave energy^2^. However, these types of power are fraught with predictability problems and depend on particular climatic conditions. Their variability essentially highlights the importance of efficient ways of storing energy. Because it can store renewable energy until it is converted into electricity using an energy conversion device, hydrogen now plays a major role in the conversation in the energy industry^3^. Proton exchange membrane fuel cells (FCs) are among these devices. PEMFCs are most preferred in certain applications such as automotive portable electronics and onsite power generation systems due to low operating temperatures, high power density and solid electrolyte^4,5^. Developing mathematical models to understand intricacies of operation principles to improve performance is one of the biggest challenges facing PEMFC technology today^6,7^. However, since the behavior of multi-physics systems, such as PEMFCs, is highly non-linear and it is difficult to establish precise models for these systems under operating conditions, proper estimation methods become indispensable for using PEMFC technology.

The application of methods called metaheuristic algorithms for parameter estimation in proton exchange membrane fuel cells (PEMFCs) has been studied in several recent studies^12,13^. Nevertheless, compared to typical gradient based approaches, contemporary metaheuristic algorithms treat problems derivative free and allow for more wide variety of problems formulation. These types of algorithms can be grouped into four categories namely: These optimization algorithms are biological, for example, Grey Wolf Optimizer^14^, physical, for instance, Multi-Verse Optimizer^15^, social, for instance, Teaching Learning-Based Optimization (TLBO)^16^and mathematical, for instance, Sine-Cosine Algorithm (SCA)^17^. Specific mechanisms of each of these metaheuristics mimic some characteristics of their original inspiration. For instance, GWO mimics the behavior of grey wolves in a chase after prey, which ranks them into hierarchical positions such as alpha, beta, delta and omega with a three stage hunting sequence: tracking, encircling and attacking. The MVO algorithm draws from the multi-verse theory in physics which states that universe creation results from massive explosions; thus the process of the algorithm includes cosmological phenomena such as wormholes, white holes as well as black holes among others^15^. On the other hand TLBO resembles what goes on inside a classroom where a teacher imparts knowledge to learners who interact with each other individually to create a good learning environment^16^. Finally, SCA employs sine and cosine mathematical functions to lead towards optimum solution thus revealing its mathematical foundation^17^.

The increased effectiveness of optimization in the estimation of PEMFC parameters through metaheuristics has been achieved by various enhancement techniques such as local search^18^, Levy flight^19^, and auxiliary operators^20^. In addition, efforts have been made to increase the accuracy of parameter estimation by combining two metaheuristic algorithms such as WCA-MFO^21^and PSO-DOX^22^.

To apply meta-heuristic algorithms and their improvements into the estimation of PEMFCs parameters, general procedure can be broken down into several steps. (1) By using datasets from manufacturers’ information as well as experimental data carried out to build a model; (2) Selecting evaluation metrics like SSE^23^, RMSE^23^and MAPE^23^which would measure accuracy in formulating objective function; (3) Constraints^23^-which also include boundaries for unknown parameters based on previous work^24,25^; (4) Control parameters for these algorithms may be adjusted appropriately^13^; (5) Initializing and iterating through themetaheuristic algorithms; (6) Checking if stopping condition is reached or not; (7) Giving those optimal values out and extracting decision variables, which are PEMFC’s parameters; finally, (8.) Parameters extracted were used to estimate V-I performance^26^. This stage demands comprehensive and accurate specification of constraint conditions needed for accurate estimations.

As promising technologies for clean energy conversion, fuel cells provide efficient and environmentally benign solutions to a variety of applications. Of all the types, Proton Exchange Membrane Fuel Cells (PEMFCs) are the most studied because of their high-power density, fast start up and low operating temperatures. Solid Oxide Fuel Cells (SOFCs) however, have become potent competitors, especially in high demand applications. SOFCs are versatile for both fuel cell and electrolyser cell operations, operating over low, intermediate and high temperature ranges. Along with this they permit the usage of broader range of fuels such as hydrogen, carbon monoxide and hydrocarbons. Gain in SOFC electrode materials’ durability, efficiency and catalytic activity as are pointed out by recent studies. For example, studies of functionalized electrode properties^27^, intermediate temperature performance^28^, and the effect of anode configurations on durability [29] highlight advances in SOFC technology^30,31^. Development of these systems demonstrate SOFCs as robust alternatives to PEMFCs in systems requiring long lifetimes and robust thermal management^32^.

Metaheuristic algorithms are highly competitive in addressing complex real-world optimization challenges, yet they often suffer from premature convergence to local optima due to limitations in their performance. The no free lunch (NFL) theorem posits that no single algorithm consistently outperforms all others across various application domains [33]^34^, . Despite this, researchers continually enhance these algorithms to develop more effective and broadly applicable optimization methods. Common enhancements include hybridizing algorithms and incorporating diverse strategic principles. While the Artificial Rabbits Optimization (ARO) has demonstrated effectiveness across various test scenarios and practical applications, it still presents opportunities for improvement, particularly in convergence accuracy, population diversity, and susceptibility to local optima. Recent advancements have led to the development of a new meta swarm intelligence optimization algorithm, named MNEARO, which integrates a mutation strategy^35^, a prey identification strategy^36^, and an elite opposition-based learning strategy (EOBLS)^37^into ARO. According to recent studies^34^, MNEARO markedly outperforms comparative algorithms in 69.2% of test functions and consistently identifies optimal solutions across different optimization challenges. This paper utilizes the MNEARO algorithm to tackle a critical research problem in the electrical engineering optimization field—the extraction of PEMFC parameters for optimal application.

This paper makes significant contributions in the following areas:

1. It employs the MNEARO algorithm to extract seven unknown parameters ( $\:{\xi\:}_{1},\:{\xi\:}_{2},\:{\xi\:}_{3},\:{\xi\:}_{4},\:\lambda\:,\:{R}_{c}\:$and *B*) of PEMFCs, addressing an electrical engineering optimization challenge.
2. The study evaluates six commercially available PEMFC stacks, namely BCS 500 W-PEM^38^, 500 W SR-12PEM^39^, Nedstack PS6 PEM^39^, H-12 PEM^40^, HORIZON 500 W PEM^40^, and a 250 W-stack [41], as the test cases.
3. A comprehensive statistical data analysis is conducted comparing MNEARO with nine other PSO variants including ARO [42], TLBO^43^, DE^35^, and SSA [44].
4. The study assesses Sum of Squared Errors (SSE), Absolute Error (AE), Relative Error (RE), and Mean Bias Error (MBE), as well as I/V and P/V characteristics across different datasheets of the selected PEMFC stacks.

The structure of this paper is methodically organized as follows: Sect. 2 introduces the mathematical model of PEMFCs; Sect. 3 details the proposed strategy; Sect. 4 discusses experimental results and related insights; and Sect. 5 presents the conclusions.

## PEMFC Modelling

This section begins by presenting a detailed semi-empirical model along with the specifications of the chosen proton exchange membrane fuel cell (PEMFC). Following this, it outlines the definition of the objective function and explores statistical comparison measures including Mean Biased Error (MBE) and the efficiency of the objective function.

In addition to their low operating temperatures, Proton Exchange Membrane Fuel Cells (PEMFCs) were selected as the standard system for this study due to their widespread application and advantageous characteristics. PEMFCs have high power density and short start up times, which make them suitable for transportation, portable electronics and stationary power generation systems. The solid polymer electrolyte in PEMFCs simplifies the system design through elimination of liquid electrolyte management and the corrosion issues present in other fuel cell types. Moreover, PEMFC’s complex nonlinear behaviors and multi-physics interactions make modeling them quite challenging.

This research uses a semi-empirical electrochemical model that is not limited to PEMFCs and can be adapted to other forms of fuel cells with appropriate modifications. This modeling approach could be extended to Solid Oxide Fuel Cells (SOFCs), Molten Carbonate Fuel Cells (MCFCs), or Alkaline Fuel Cells (AFCs), by adjusting model parameters and accounting for various electrochemical reactions, electrolyte materials, and operating conditions. This adaptability then points to the broader applicability of the model and optimization strategies to a wider set of fuel cell technologies, thereby enhancing the powerful utility of the optimization to multiple types of systems.

### Semi-empirical electrochemical model

The output voltage of the FC stack ($\:{V}_{fc})$ is obtained using Eqs. (1),

1 $${V}_{fc}=({V}_{Nernst}-{V}_{act}-{V}_{ohmic}-{V}_{con})\cdot{N}_{cell}$$

In this context, $\:{V}_{act}$ denotes the activation polarization, resulting from the slow reaction rates at the electrode surface. $\:{V}_{ohmic}$​ refers to ohmic polarization, which accounts for resistance encompassing all electrical and ionic conduction losses through the electrolyte, catalyst layers, cell interconnects, and contacts. $\:{V}_{con}$​ signifies concentration polarization, which relates to the disparity in concentration between the fuel/air channel and the chemical species present on the electrode surface, while $\:{N}_{cell}$​ represents the total number of cells^45^. The reversible cell voltage, known as Nernst voltage $\:{V}_{Nernst}$, can be calculated using Eq. (2)^46,47^.

2 $$\:{V}_{Nernst}=-1.229-0.85 \times\:{10}^{-3}({T}_{stack}-298.15)+4.3085\times\:{10}^{-5}{T}_{stack}\left[ln\right({p}_{{H}_{2}})+0.5ln({p}_{{O}_{2}}\left)\right]$$

In this formulation, $\:{T}_{stack}$​ refers to the stack temperature measured in Kelvin (K), $\:{p}_{{H}_{2}}$ indicates the partial pressure of hydrogen in bar, and $\:{p}_{{O}_{2}}$​​ is the partial pressure of oxygen, also in bar. The partial pressure of hydrogen is determined using Eq. (3)^46^.

3 $$\:{p}_{{H}_{2}}=0.5 \cdot R{H}_{a} \cdot{P}_{{H}_{2}O}^{sat}[{\left(\text{e}\text{x}\text{p}\right(\frac{1.635\left(\frac{{I}_{fc}}{{A}_{cell}}\right)}{{T}_{stack}^{1.334}})\times\:\frac{R{H}_{a} \cdot{P}_{{H}_{2}O}^{sat}}{{P}_{a}})}^{-1}-1]$$

If the pure oxygen is fed to the cathode side of the FC, the partial pressure of oxygen at the cathode can be calculated using Eq. (4).

4 $$\:{p}_{{O}_{2}}={P}_{c}-\left(R{H}_{c} \cdot{P}_{{H}_{2}O}^{sat}\right) \cdot [{\left(\text{e}\text{x}\text{p}\right(\frac{4.192\left(\frac{{I}_{fc}}{{A}_{cell}}\right)}{{T}_{stack}^{1.334}})\cdot\frac{\left(R{H}_{c}\cdot{P}_{{H}_{2}O}^{sat}\right)}{{P}_{c}})}^{-1}-1]$$

If the air is used instead of pure oxygen, the partial pressure of oxygen at the cathode can be calculated using Eq. (5).

5 $$\:{p}_{{O}_{2}}={P}_{c}-(R{H}_{c}\cdot{P}_{{H}_{2}O}^{sat})-\frac{0.79}{0.21}\cdot{p}_{{O}_{2}}\cdot\text{e}\text{x}\text{p}\left(\frac{0.291\left(\frac{{I}_{fc}}{{A}_{cell}}\right)}{{T}_{stack}^{0.832}}\right)$$

where $\:R{H}_{a}$ and $\:R{H}_{c}$ are relative humidity of vapors in the anode and cathode, respectively. $\:{I}_{fc}$ is the FC operating current (A), $\:{A}_{cell}$ is the active cell area (cm2), $\:{P}_{a}$ is the anode pressure (bar), and $\:{P}_{c}$ is the cathode pressure (bar). $\:{P}_{{H}_{2}O}^{sat}$is the saturation pressure of the water vapor (bar) and can be calculated as a function of the stack temperature using Eq. (6)^46, 47^.

6 $$\:{\text{l}\text{o}\text{g}}_{10}\left({P}_{{H}_{2}O}^{sat}\right)=2.95\times\:{10}^{-2}({T}_{stack}-273.15)-9.18\times\:{10}^{-5}{({T}_{stack}-273.15)}^{2}+1.44\times\:{10}^{-7}{({T}_{stack}-273.15)}^{3}-2.18$$

The activation polarization can be calculated depending on the stack temperature and oxygen concentration with Eq. (7)^46^,

7 $$\:{V}_{act}=-[{\xi\:}_{1}+{\xi\:}_{2}\cdot{T}_{stack}+{\xi\:}_{3}\cdot{T}_{stack}\text{l}\text{n}({C}_{{O}_{2}})+{\xi\:}_{4}\cdot{T}_{stack}\cdot\text{l}\text{n}({I}_{FC}\left)\right]$$

where $\:{\xi\:}_{k}(k=\text{1,2},\text{3,4})$are the semi-empirical coefficients based on theoretical equations with kinetic, thermodynamic, and electrochemical foundations^48^, and $\:{C}_{{O}_{2}}$ is the oxygen concentration (mol $\cdot$cm−3) that can be calculated using Eq. (8)^46^.

8 $$\:{C}_{{O}_{2}}=\left(\frac{{p}_{{O}_{2}}}{5.08}\right)\times\:{10}^{6}\text{e}\text{x}\text{p}(-\frac{498}{{T}_{stack}})$$

The ohmic polarization depends on the membrane resistance, $\:{R}_{m}$ (Ω), and contact resistance, $\:{R}_{C}$ (Ω), as given in Eq. (9) [46].

9 $$\:{V}_{ohmic}={I}_{FC}\cdot({R}_{m}+{R}_{C})$$

The membrane resistance depends on the resistivity of the membrane, $\:{\rho\:}_{m}$ (Ω.cm), membrane thickness, $\:l$ (cm), and effective membrane area (cm2), which is shown in Eq. (10).

10 $$\:{R}_{m}=\frac{{\rho\:}_{m}l}{{A}_{cell}}$$

The membrane resistivity ($\:{\rho\:}_{m}$) is calculated by using Eq. (11) for Nafion membranes.

11 $$\:{\rho\:}_{m}=\frac{181.6[1+0.03(\frac{{I}_{fc}}{{A}_{cell}})+0.062{\left(\frac{{T}_{stack}}{303}\right)}^{2}{\left(J\right)}^{2.5}]}{[\lambda\:-0.643-3(\frac{{I}_{fc}}{{A}_{cell}}\left)\right]\text{e}\text{x}\text{p}\left(4.18\right(\frac{{T}_{stack}-303}{{T}_{stack}}\left)\right)}$$

where $\:\lambda\:$is an adjustable parameter related to the membrane and its preparation process^48^. The concentration polarization is calculated using Eq. (12)^46^.

12 $$\:{V}_{con}=-\beta\:\text{l}\text{n}(1-\frac{J}{{J}_{max}})$$

where $\:\beta\:$ is the parametric coefficient (V) that depends on the cell and its operation state [46], $\:J$ is the actual current density (A $\cdot$ cm − 2), and $\:{J}_{max}$ is the maximum current density (A $\cdot$ cm − 2).

### Fitness function definition

In this research, various versions of Particle Swarm Optimization (PSO) and Fractional Differential Evolution (FD-DE) are employed to refine the model parameters of the proton exchange membrane fuel cell (PEMFC). This optimization aligns the model outcomes with established data from the literature or manufacturer specifications, thereby improving the model’s accuracy. The output voltage is determined at specific points correlating with each current value using the mathematical models detailed in the Section on Semi-empirical Electrochemical Models. Consequently, the proposed fitness function, which assesses the quality of the estimated parameters, utilizes the Sum of Squared Errors (SSE) defined in Eq. (13) as the fitness function [46].

13 $$\:SSE=Min\left(\sum\:_{i=1}^{N}{\left[{V}_{meas}\right(i)-{V}_{calc}(i\left)\right]}^{2}\right)$$

In this context, $\:N$ represents the total number of measured data points, $\:i$ is the iteration counter, $\:{V}_{meas}$​ refers to the measured voltage of the fuel cell, and $\:{V}_{calc}\:$indicates the calculated voltage of the fuel cell. Various Multi-Attribute Decision Making (MADM) methods, each based on different principles, are detailed in the section titled “Ranking of the Algorithms.” These methods are applied to determine the most effective Metaheuristic Algorithms (MHAs) for the H-1000 XP case study. The Mean Biased Error (MBE) is computed using Eq. (14).

14 $$\:MBE=\frac{{\sum\:}_{i=1}^{N}\left|{V}_{meas}\right(i)-{V}_{calc}(i\left)\right|}{N}$$

In optimization process, the fitness function is a critical component, acting as the quantitative measure that directs our algorithms towards the optimal set of PEMFC parameters. The fitness function as the Sum of Squared Errors (SSE) of the measured voltages ($\:{V}_{\text{meas}}$) and the calculated voltages ($\:{V}_{\text{calc}}$), in order to minimize the overall discrepancy between the experimental data and the model predictions. This method guarantees that the resulting optimized parameters produce a model that closely matches the actual behavior of the fuel cell over a range of operating conditions.

The choice of SSE as the fitness function is significant for several reasons:

1. **Sensitivity to Large Errors**: SSE squares errors, this means bigger differences between observed and predicted values are more important, forcing the optimization algorithm to concentrate on decreasing important disparities that might seriously dent the accuracy of the model.
2. **Robustness in Parameter Estimation**: The SSE allows us to get a more comprehensive measure of how the model performs for all data points, and provides a more robust parameter estimation than the isolated data points approach, as it takes into account the collective behavior of the system.
3. **Standardization and Comparability**: The use of SSE ensures consistency with other PEMFC modeling studies and methodologies, permitting direct comparisons and validations against results in the literature.
4. **Mathematical Convenience**: The function SSE is a differentiable and continuous function, which is beneficial for the convergence properties of the optimization algorithms, MNEARO and others discussed in this study.

The optimization algorithms use the PEMFC parameters iteratively to minimize the SSE while adjusting the parameters in such a manner so that the model comes closer as possible with the empirical data. This is a key step in improving the model’s predictive capabilities, which is critical to the simulation, control, evaluation, management, and optimization of PEMFC systems.

## Mutational Northern Goshawk and Elite opposition learning-based Artificial Rabbits Optimizer (MNEARO)

### Standard ARO

In order to protect their nests from attacks, Artificial Rabbits often forage near the nesting site of other rabbits. Every AR digs several burrows around its nest, randomly selects one to hide in and thus vastly improves its chances for survival^48^. Because of the high number of predators that prey on ARs in the wild, these animals must alternate between roundabout foraging and random hiding depending on their condition. With this regard, we define in this section a mathematical model that describes how ARs practice survival techniques.

### Initialization

For SIAs, the initial population is usually obtained by random initialization, and the matrix can be expressed as:

15 $$\begin{array}{c}RAs=\left[\begin{array}{ccccc}{\text{Individual}}_{\text{1,1}}&\:{\text{Individual}}_{\text{1,2}}&\:\cdots\:&\:\cdots\:&\:{\text{Individual}}_{1,d}\\\:{\text{Individual}}_{\text{2,1}}&\:{\text{Individual}}_{\text{2,2}}&\:\cdots\:&\:\cdots\:&\:{\text{Individual}}_{2,d}\\\:\dots\:&\:\dots\:&\:\cdots\:&\:\cdots\:&\:\cdots\:\\\:\dots\:&\:\dots\:&\:\cdots\:&\:\cdots\:&\:\cdots\:\\\:{\text{Individual}}_{n,1}&\:{\text{Individual}}_{n,2}&\:\cdots\:&\:\cdots\:&\:{\text{Individual}}_{n,d}\end{array}\right]\text{,}\end{array}$$

Where, $\:d\:$is the individual dimension and $\:n$ is the population size. The formula for random initialization is as follows:

16 $$\:\begin{array}{c}{\text{Individual}}_{i,j}=L{b}_{j}+\left(U{b}_{j}-L{b}_{j}\right)\times\:\text{r}\text{a}\text{n}\text{d}\end{array}$$

‘$\:rand{\prime\:}$ here is a random number between $\:\left(\text{0,1}\right).L{b}_{j}$ and $\:U{b}_{j}$ are the lower bound and upper bound of $\:j-th$ dimension respectively. $\:i$ and $\:j$ are indices ranging from 1 to $\:n$ and 1 to $\:d$ respectively, indicating how far each dimension extends on the model.

### Detour foraging

When foraging, Artificial Rabbits (AR) will bypass food sources close to their own nests and instead randomly approach another AR’s nest to forage. This is known as detour foraging in ARO, which contains two separate moves. Initially, the $\:i-th$ chosen AR takes forward steps by using a selected random $\:j-th$ AR and step vector $\:D$. Then the second behavior augments AR’s path randomness by introducing higher position perturbations. A detailed formula of this detour foraging process is given below.

17 $${\mathbf{m}}_{\mathbf{i}}(t+1)={\mathbf{x}}_{\mathbf{j}}\left(t\right)+D\cdot\:\left({\mathbf{x}}_{\mathbf{i}}\left(t\right)-{\mathbf{x}}_{\mathbf{j}}\left(t\right)\right)+round\left(0.5\cdot\:\left(0.05+{r}_{1}\right)\right)\cdot\:{N}_{1}$$

18 $$D=dis\cdot\:ch$$

19 $$dis=\left(e-{e}^{{\left(\frac{ \llcorner-1}{T}\right)}^{2}}\right)\cdot\:sin\left(2\pi\:\cdot\:{r}_{2}\right)$$

20 $$\:ch\left(k\right)=\left\{\begin{array}{c}1\text{i}\text{f}k=\mathbf{g}\left(s\right)\\\:\text{0 else}\end{array},k=\text{1,2},\cdots\:,d\text{a}\text{n}\text{d}s=\text{1,2},\cdots\:, \lceil{r}_{3}\cdot\:d \rceil\right.$$

21 $$g=randperm\left(d\right)$$

In this expression, $\:{\varvec{m}}_{i}(t+1)$ denotes the candidate position of the $\:i-th$ AR in the $\:(t+1)-th$ generation. $\:{\varvec{x}}_{i}\left(t\right)$ and $\:{\varvec{x}}_{j}\left(t\right)$ represent the positions of the $\:i-th$ AR and a randomly selected AR in the $\:t-th$ generation, respectively, where $\:i,j=\text{1,2},\cdots\:,n,i\ne\:j.\:$ The variables $\:t$ and $\:T$ refer to the current iteration and the maximum number of iterations, respectively. The function $\:round\left(\:\right)$ computes the rounded value, while $\:\text{r}\text{a}\text{n}\text{d}\text{p}\text{e}\text{r}\text{m}\left(d\right)$ generates an array of integers from $\:[1,d]$ in a random order. $\:{r}_{1},{r}_{2},{r}_{3}$ are three random numbers within the range (0,1), and $\:{N}_{1}\:$is a random number drawn from the standard normal distribution.

As iterations progress, the variable $\:dis$ tends to stabilize, indicating that the population quality is sufficiently high in later iterations to shift the algorithm’s focus from exploration to exploitation. Meanwhile, $\:ch$ randomly selects a specific number of dimensions to update, thereby enhancing the randomness and effectiveness of the exploration phase.

### Random hiding

During each iteration of ARO, each Artificial Rabbit (AR) creates a set number of burrows around its nest based on its individual dimension and randomly selects one of these burrows for hiding. This random selection strategy enhances the AR’s chances of survival if detected by predators. The mathematical model that describes the process of burrow creation is outlined below:

22 $$\:\begin{array}{c}{\mathbf{B}}_{\mathbf{i},\text{j}}\left(t\right)={\mathbf{x}}_{\mathbf{i}}\left(t\right)+h\cdot\:v\cdot\:{\mathbf{x}}_{\mathbf{i}}\left(t\right)\end{array}$$

23 $$\text{h}=\frac{T-t+1}{T}\cdot{N}_{2}$$

24 $$\:v\left(k\right)=\left\{\begin{array}{cc}1&\:\text{if} \;k=j\\\:0&\:\text{else}\end{array},k=\text{1,2},\cdots\:,d\right.$$

In this model, $\:{\varvec{B}}_{i,j}\left(t\right)$ represents the $\:j-th$ burrow created by the $\:i-th$ AR in generation $\:t$, where iii ranges from 1 to $\:n$ and $\:j$ from 1 to $\:d$. The term $\:h$ is defined as the hiding coefficient, and $\:{N}_{2}$​ is a random number drawn from the standard normal distribution.

To enhance their chances of survival, each AR must randomly select one of its burrows as a shelter to protect itself from predators. The equation for this random selection process is presented below:

25 $$\:\begin{array}{c}{\mathbf{m}}_{\mathbf{i}}(t+1)={\mathbf{x}}_{\mathbf{i}}\left(t\right)+D\cdot\:\left({r}_{4}\cdot\:{\mathbf{B}}_{\mathbf{i},\mathbf{r}}\left(t\right)-{\mathbf{x}}_{\mathbf{i}}\left(t\right)\right)\end{array}$$

Here, $\:{B}_{i,r}\left(t\right)$ denotes the burrow randomly chosen by the $\:i-th$ AR in the $\:t-th$ generation, where $\:i$ ranges from $\:1$ to $\:n$ and $\:r$ from $\:1$ to $\:d$. The variable $\:{r}_{4}$​ represents a random number between 0 and 1.

### Energy shrink

Below is the pseudocode for Artificial Rabbits Optimization (ARO) as shown in Algorithm 1. The method is based on AR’s physical energy gradual decrease with time and involves detour foraging at the beginning of the iteration process and random hiding later. To calculate this conversion, we need an energy factor that contains which is important:

26 $$\:\begin{array}{c}E=4\left(1-\frac{t}{T}\right)ln\frac{1}{{r}_{5}}\end{array}$$

The ARO algorithm utilizes a greedy selection mechanism to finalize the population, and the formula for this selection process is specified as follows:

27 $$\:\begin{array}{c}{\mathbf{x}}_{\mathbf{i}}(t+1)=\left\{\begin{array}{cc}{\mathbf{x}}_{\mathbf{i}}\left(t\right)&\:f\left({\mathbf{x}}_{\mathbf{i}}\left(t\right)\right)\le\:f\left({\mathbf{m}}_{\mathbf{i}}(t+1)\right)\\\:{\mathbf{m}}_{\mathbf{i}}(t+1)&\:f\left({\mathbf{x}}_{\mathbf{i}}\left(t\right)\right)>f\left({\mathbf{m}}_{\mathbf{i}}(t+1)\right)\end{array}\right.\end{array}$$

Where $\:($ ) is the fitness function corresponding to the optimization problem.

### A new variant of ARO (MNEARO)

**Fig. 1:**
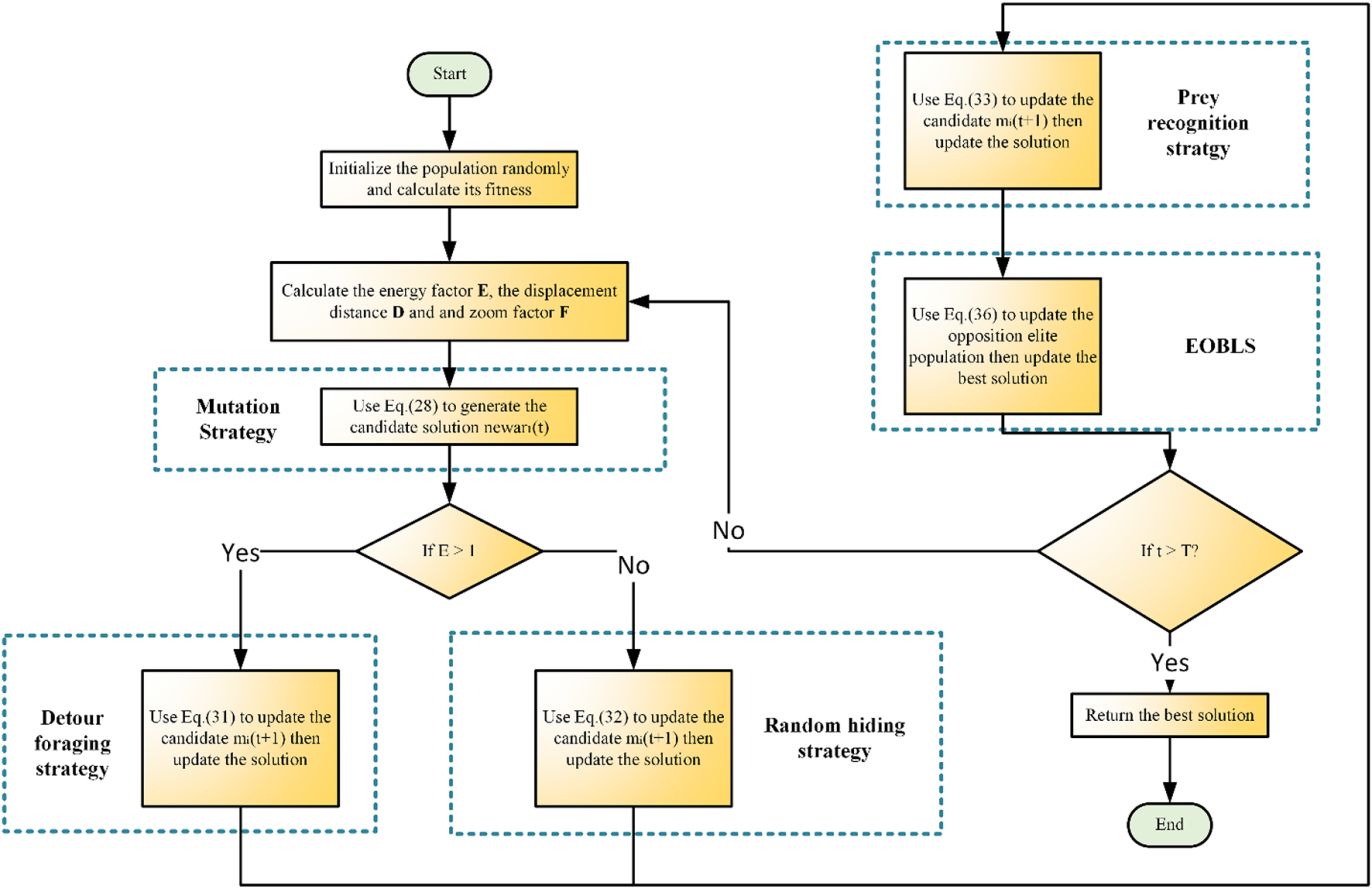
The flow chart of MNEARO.

It is possible that ARO might only be trapped by the local optima because of its limitation in terms of optimization. This happens even though ARO through energy reduction successfully balances exploration and exploitation and adopts natural behaviours. Consequently, to solve such problems, this section presents a more developed version of ARO referred as MNEARO (see Fig. 1).

### Mutation strategy

It has been there for so long and even now Differential Evolution (DE) is still a beloved algorithm across different scientist disciplines because of its simplicity and efficiency. DE makes use of the same update stage as Genetic Algorithms (GA) but usually outperforms them due to variations in execution sequence and associated mathematical formulations. In this example, the mutation formula from DE that enhances randomness in the algorithm is combined with two internal processes from Artificial Rabbits Optimization (ARO) that enhance its exploration ability. The mutation formula employed in ARO is presented below:

28 $$\:\mathbf{n}\mathbf{e}\mathbf{w}\mathbf{a}{\mathbf{r}}_{\mathbf{i}}\left(\mathbf{t}\right)={\mathbf{x}}_{p1}\left(t\right)+F\cdot \left({\mathbf{x}}_{p2}\left(t\right)-{\mathbf{x}}_{p3}\left(t\right)\right)$$

29 $$\:\begin{array}{c}F={F}_{0}\cdot\:{2}^{a}\end{array}$$

30 $$\text{a}={e}^{1-\frac{T}{T+1-t}}$$

Where $$p1,p2,p3\in\:\text{1,2},\cdots\:,n,i\ne\:p1\ne\:p2\ne\:p3,\:{F}_{0}=0.5.$$

**Algorithm 1:**
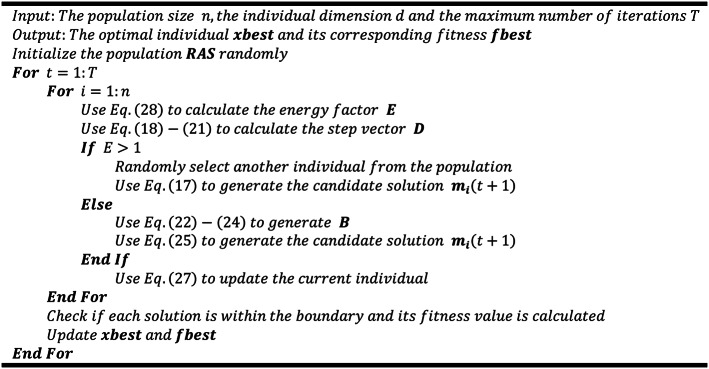
ARO

31 $$\:\begin{array}{c}{\mathbf{m}}_{\mathbf{i}}\left(t+1\right)={\text{newar}}_{\mathbf{i}}+D\cdot\:\left({\mathbf{x}}_{i}\left(t\right)-{\text{newar}}_{\mathbf{i}}\right)+\text{round}\left(0.5\cdot\:\left(0.05+{r}_{1}\right)\right)\cdot\:{N}_{1}\end{array}$$

32 $${\mathbf{m}}_{\mathbf{i}}\left(t+1\right)={\text{newar}}_{\mathbf{i}}+D\cdot\:\left({r}_{4}\cdot\:{\mathbf{B}}_{\mathbf{i}\mathbf{r}}\left(t\right)-{\text{newar}}_{\mathbf{i}}\right)$$

The detour foraging and random hiding behaviors in ARO have been enhanced by incorporating a mutation formula, allowing individuals to recalibrate positions based on three others, improving exploration and avoiding local optima. These modifications ensure sustained randomness and better solution space exploration in later iterations.

### Prey identification strategy

This section enhances ARO by incorporating predator-like strategies from NGO, focusing on prey identification to boost exploration while preserving ARO’s strong exploitation ability. Experimental results show that integrating this strategy enhances ARO’s performance without compromising its original effectiveness. The prey identification formula adopted in ARO is detailed below:

33 $$\:\begin{array}{c}{\mathbf{m}}_{\mathbf{i}}(t+1)=\left\{\begin{array}{c}{\mathbf{x}}_{\mathbf{i}}\left(t\right)+{r}_{6}\times\:\left({\mathbf{x}}_{\mathbf{k}}\left(t\right)-I\cdot\:{\mathbf{x}}_{\mathbf{i}}\left(t\right)\right)\:{f}_{k}<{f}_{i}\\\:{\mathbf{x}}_{\mathbf{i}}\left(t\right)+{r}_{6}\times\:\left({\mathbf{x}}_{\mathbf{i}}\left(t\right)-{\mathbf{x}}_{\mathbf{k}}\left(t\right)\right)\:{f}_{k}\ge\:{f}_{i}\end{array}\right.\end{array}$$

In this context, $\:i,k=\text{1,2},\cdots\:,n\:$and $\:i\ne\:k$. The terms $\:{f}_{i}$​ and $\:{f}_{k}$​ represent the fitness values of the $\:i-th$ and $\:k-th$ individuals, respectively. The variable $\:{r}_{6}$​ is a random number between 0 and 1, while III is a random number that can either be 1 or 2.

To update the population, ARO uses Eq. (31) and Eq. (32) to generate candidate populations. To improve ARO’s optimization capabilities, the prey identification formula from NGO is employed as the update mechanism for any Artificial Rabbit (AR) that fails to escape, thereby creating corresponding candidate individuals.

### Elite opposition-based learning strategy

ARO enhances exploitation by allowing elite individuals with top fitness to learn from one another, reducing reliance on the current optimal solution and avoiding local optima. The Elite Opposition-Based Learning Strategy (EOBLS) [49] generates an opposition elite population (OEX) to explore the solution space, combining it with the original population through greedy selection. The formulae for calculating elite and opposition elite individuals are provided below in Eq. 34 to 38

34 $${\mathbf{E}\mathbf{X}}_{\mathbf{i}}^{\mathbf{t}}=\left[e{x}_{i,1}^{t},e{x}_{i,2}^{t},\cdots\:,e{x}_{i,d}^{t}\right]$$

35 $${\mathbf{O}\mathbf{E}\mathbf{X}}_{\mathbf{i}}^{\mathbf{t}}=\left[oe{x}_{i,1}^{t},oe{x}_{i,2}^{t},\cdots\:,oe{x}_{i,d}^{t}\right]$$

36 $$oe{x}_{i,j}^{t}={r}_{7}\cdot\:\left(e{l}_{j}^{t}+e{u}_{j}^{t}\right)-e{x}_{i,j}^{t}$$

37 $$e{l}_{j}^{t}=min\left(e{x}_{k,j}^{t}\right)$$

38 $$e{u}_{j}^{t}=max\left(e{x}_{k,j}^{t}\right)$$

where $\:i,k=\text{1,2},\cdots\:,n/10,j=\text{1,2},\cdots\:,d.\varvec{e}{\varvec{l}}^{t}$ and $\:\varvec{e}{\varvec{u}}^{t}$ are the lower and upper bound vectors of the elite population in the $\:t$ th generation respectively.

Assessing the computational complexity of MNEARO is essential for understanding its operational efficiency. The components contributing to this evaluation include population initialization $\:O\left(\text{P}\text{I}\right)$, detour foraging following mutation $\:O\left(\text{M}\text{D}\text{F}\right)$, random hiding post-mutation $\:O\left(\text{M}\text{R}\text{H}\right)$, the prey identification formula $\:O\left(\text{P}\text{R}\text{F}\right),$ and the elite opposition-based learning strategy $\:O$ (EOBLS). The detailed steps for this calculation are as follows:

39 $$\:O\left(\text{MNEARO}\right)=O\left(\text{P}\text{I}\right)+O\left(\text{M}\text{D}\text{F}\right)+O\left(\text{M}\text{R}\text{H}\right)+O\left(\text{P}\text{R}\text{F}\right)+O\left(\text{EOBLS}\right)==O\left(nd+2Td+3.2\text{Tnd}+0.5\text{Tnd}{d}^{2}\right)$$

**Algorithm 2:**
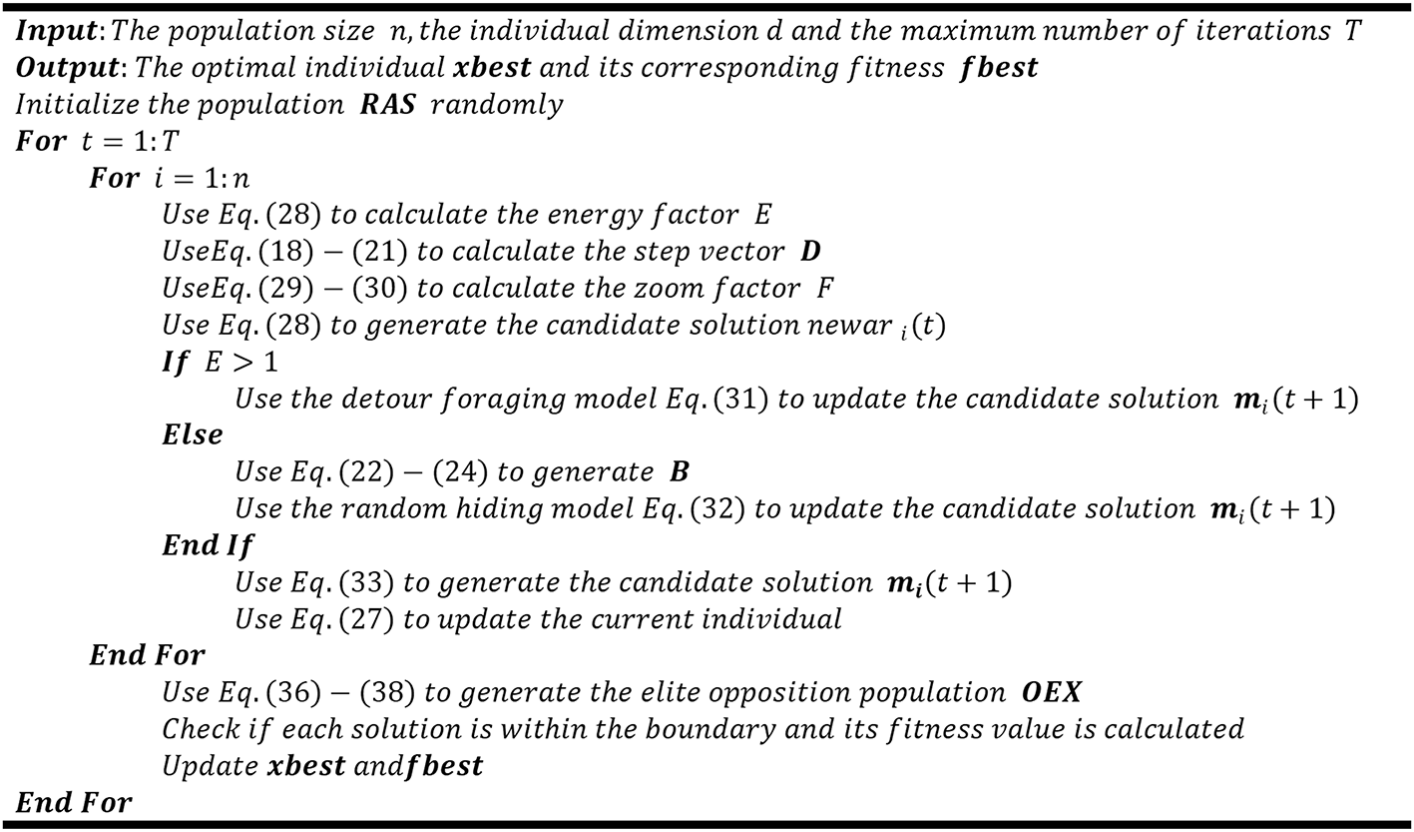
MNEARO

## Result analysis

In this work an attempt has been made to exhaustively illustrate MNEARO algorithm and compare it with different optimization algorithms like, ARO[42], TLBO^43^, DE^35^and SSA [44] applied for PEMFC modelling. The default parameter settings for different algorithms used in literatures^34^are given in Table 1. All algorithms compared were set to their recommended to estimate the parameter of a PEMFC fuel cell (BCS 500 W- PEMFC^38^, 500 W SR-12 PEMFC^39^, Nedstak PS6 PEMFC^39^, H-12 PEMFC^40^, HORIZON 500 W PEMFC^40^ and 250 W-stack PEMFC [41]) presented in Table 2. All the experiments are carried out on Matlab 2021a of a PC with Windows Server 2019 operating system CPU i7-11700k@3.6 GHz, maximum iterations 500, number of run 50 and population size 40.

**Table 1:** Default parameter settings of the compared algorithms.

Algorithms	Default settings
ARO	*N* = 100, *T* = 500, *m* = 30
DE	$\:VR=0.5,\:CR=0.9$
TLBO	The value in TF is randomly selected 1 or 2
SSA	$\:PR=0.2,\:DR=0.1,\:ST=0.8$
MNEARO	*N* = 100, *T* = 500, *m* = 30

**Table 2:** Characteristics of twelves PEMFCs used in this work.

S. No.	PEMFC Type	Power(W)	Ncells (no)	A(cm^2^)	l(um)	T(K)	Jmax(mA/cm^2^)	PH_2_(bar)	PO_2_(bar)
FC1	BCS 500 W	500	32	64	178	333	469	1.0	0.2095
FC2	NetStack PS6	6000	65	240	178	343	1125	1.0	1.0
FC3	SR-12	500	48	62.5	25	323	672	1.47628	0.2095
FC4	H-12	12	13	8.1	25	323	246.9	0.4935	1.0
FC 5	STD	250	24	27	127	343	860	1.0	1.0
FC 6	Horizon	500	36	52	25	338	446	0.55	1.0

### FC1: BCS 500 W

**Fig. 2:**
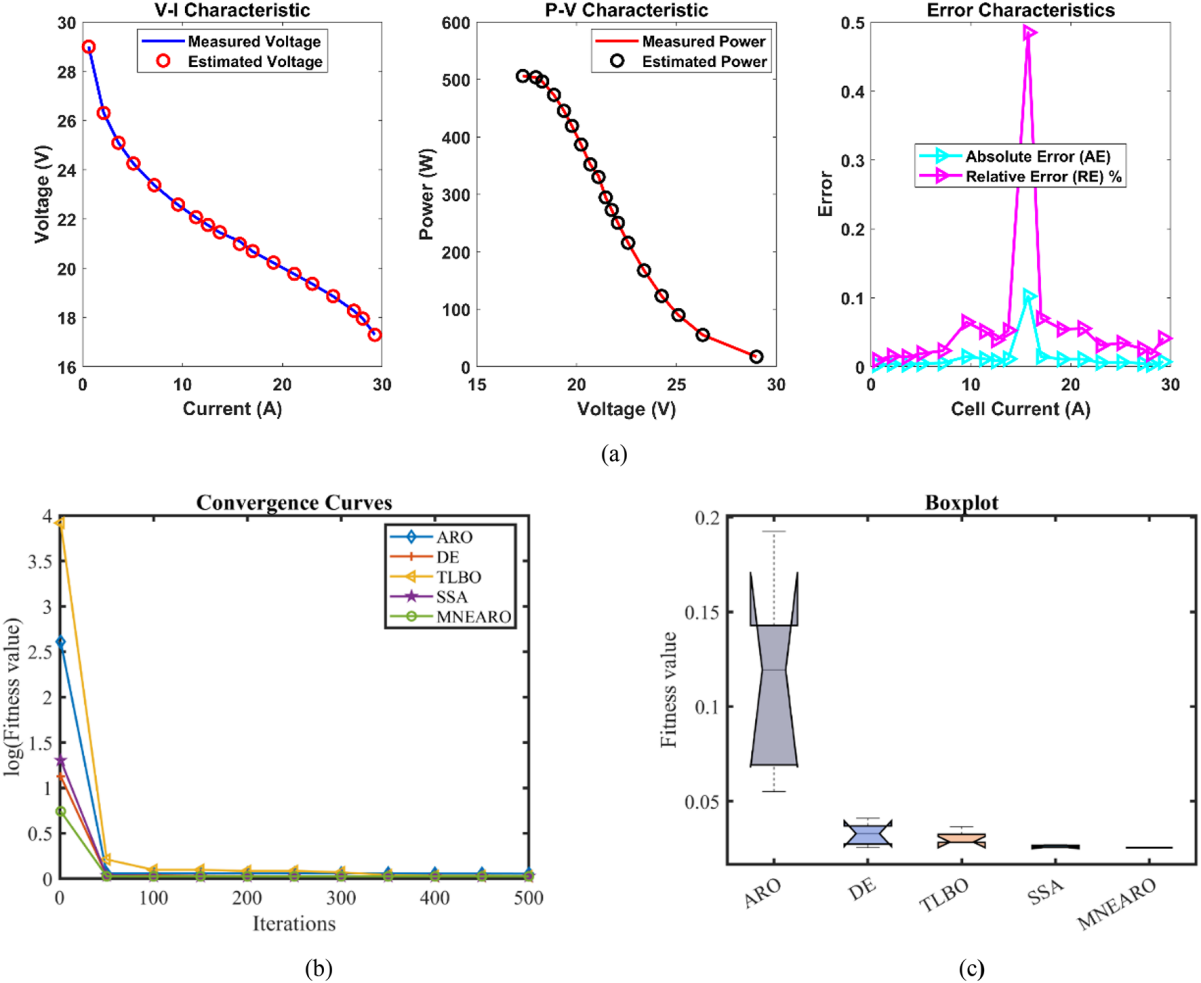
FC1 (**a**) V-I, P-V and Error Curve, (**b**) Convergence Curve, (**c**) Box-Plot.

The exceptional precision, stability, and efficiency of MNEARO are highlighted in Table 3 where it consistently achieves the lowest or near-lowest values. The minimal value for MNEARO is 0.0254927 which equals DE showing that it can repeatedly attain optimal solutions (tie with DE). Its maximum value is 0.0254928 which is significantly lower than that of ARO (0.1924899) and TLBO (0.0364916), indicating the ability to avoid high-error scenarios (as shown by a low number). According to Table 3, MNEARO has the lowest mean value of 0.0254927, which confirms its accuracy on consistent basis. Another significant measure of stability is standard deviation that has strictly been considered as negligibly small at 4.59E-08 compared to ARO’s variability (0.053443) and DE’s variability (0.0061464) that demonstrates how much more precise it is. MNEARO runs faster than DE (8.7900488 s) and SSA (6.3546924 s); thus making it the most efficient algorithm in terms of computational cost as shown by runtime RT = 2.9671325 s in Table 3. Furthermore, not only does this figure indicate optimal outputs through minimal computation but also proves its superiority over others in terms of constancy as well as effectiveness especially those under consideration for use in highly accurate applications with time limits. Tables 3 and 4; Fig. 2 show us that MNEARO gives best results without using up too much processing time while being constantly better than all other examined algorithms on indicators like stability or efficiency.

**Table 3:** Optimized parameters and optimal function value for FC1.

Algorithm	ARO	DE	TLBO	SSA	MNEARO
$\:{\varvec{\xi\:}}_{1}$	-0.9840126	-0.8721622	-1.1105925	-1.1504024	-0.9259692
$\:{\varvec{\xi\:}}_{2}$	0.00301	0.0022555	0.0031004	0.0036846	0.0026293
$\:{\varvec{\xi\:}}_{3}$	6.454E-05	3.718E-05	4.552E-05	7.538E-05	5.084E-05
$\:{\varvec{\xi\:}}_{4}$	-0.0001814	-0.000193	-0.0001908	-0.0001928	-0.000193
$\:\varvec{\lambda\:}$	20.681348	20.877275	21.252609	22.094647	20.877243
$\:{\varvec{R}}_{\varvec{c}}$	0.0007508	0.0001	0.0003099	0.0002184	0.0001
***B***	0.0136	0.0161261	0.0151479	0.0161511	0.0161261
***Min.***	0.0550084	0.0254927	0.0280372	0.0256024	0.0254927
***Max.***	0.1924899	0.0410172	0.0364916	0.0270263	0.0254928
***Mean***	0.1133755	0.0325788	0.0304626	0.0261257	0.0254927
***Std.***	0.053443	0.0061464	0.0035903	0.0006616	4.587E-08
***RT***	4.5317695	8.7900488	3.5392382	6.3546924	2.9671325
***FR***	5	3	3.6	2.2	1.2

The results for Case FC1 in the provided data achieved lowest minimum value of 0.0254927 for MNEARO, which was equal to the performance of DE and slightly better than other algorithms. ARO, TLBO, and SSA were outperformed by MNEARO by 53.68%, 9.08%, and 0.43%, respectively. The value of MNEARO was 0.0254928, which is the lowest among all algorithms. MNEARO outperformed ARO, DE, TLBO, and SSA by 86.75%, 37.85%, 30.14%, and 5.67%, respectively. In MNEARO, the best result was obtained with the lowest mean value of 0.0254927. The mean value of this was 77.52%, 21.77%, 16.31% and 2.42% lower than the mean values of ARO, DE, TLBO, and SSA, respectively. The MNEARO showed the highest stability with a standard deviation of 4.587E-08. The standard deviation of MNEARO compared to ARO, DE, TLBO, and SSA were 99.91%, 98.60%, 98.72%, and 99.93% lower, respectively. MNEARO was the most efficient, with a runtime of 2.9671325. The MNEARO was 34.55%, 66.24%, 16.16%, and 53.31% faster than ARO, DE, TLBO, and SSA, respectively. The Friedman rank for MNEARO was 1.2, which was the best and significantly better than other algorithms. MNEARO’s Friedman rank was 76.00%, 60.00%, 66.67%, and 45.45% better than ARO, DE, TLBO, and SSA, respectively.

**Table 4:** Performance metrics of MNEARO Algorithm for FC1.

S. NO.	V_est_ (V)	*P*_est_ (W)	AE_v_ (A)	RE %	MBE
1	28.9972224	17.3983334	0.00277763	0.00957804	4.28624E-07
2	26.3059406	55.2424753	0.00405938	0.01542905	9.15477E-07
3	25.0935607	89.8349473	0.0035607	0.01419172	7.04367E-07
4	24.254627	123.213505	0.00462701	0.01908046	1.1894E-06
5	23.375424	167.60179	0.005424	0.02320926	1.63443E-06
6	22.584624	215.683159	0.01462397	0.06479382	1.18811E-05
7	22.071337	250.509675	0.01133699	0.05139161	7.14041E-06
8	21.7584734	272.851257	0.00847343	0.03895831	3.98884E-06
9	21.4612728	294.663276	0.01127284	0.05255404	7.05983E-06
10	20.9877523	330.137344	0.10224769	0.48481599	0.000580811
11	20.6945205	352.220738	0.01452047	0.07021503	1.17136E-05
12	20.2309975	386.614362	0.01099748	0.05438911	6.71914E-06
13	19.7709551	419.144249	0.01095514	0.05544097	6.6675E-06
14	19.3660369	445.418848	0.00603688	0.03118223	2.02466E-06
15	18.8664788	473.171287	0.00647876	0.03435184	2.3319E-06
16	18.2747333	496.524504	0.0047333	0.02590751	1.24467E-06
17	17.9533236	503.77026	0.0033236	0.0185159	6.13686E-07
18	17.2928898	505.989956	0.00711019	0.04109936	2.8086E-06

### FC2: NetStack PS6

**Fig. 3:**
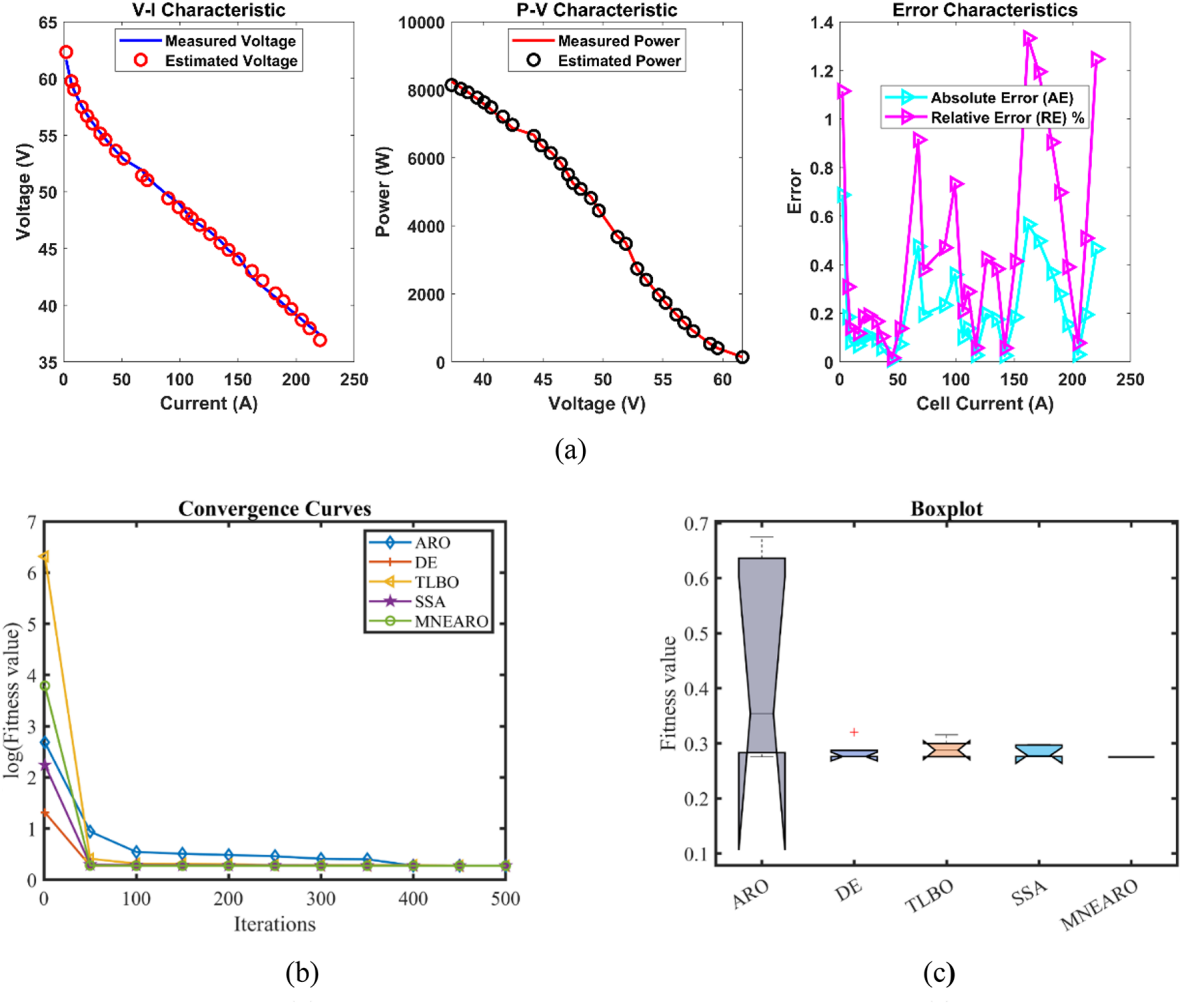
FC2 (**a**) V-I, P-V and Error Curve, (**b**) Convergence Curve, (**c**) Box-Plot.

MNEARO’s minimum value in Table 5 is 0.2752105, which is the smallest of all algorithms and indicates that it can consistently achieve the most optimal solutions. MNEARO’s maximum value is also 0.2752105, which is much lower than ARO (0.6747868) and TLBO (0.3155648), showing how robust it is to prevent high-error scenarios. Moreover, the average of MNEARO has slightly more value than any other algorithm at 0.2752105; hence it consistently produces accurate results every time. Also, this algorithm presents unrivaled stability as shown by its standard deviation close to zero (2.51E-16) compared to those of ARO (0.1917348) and DE (0.0199925), bringing out its unmatched accuracy.As far as computation efficiency is concerned, MNEARO achieves a competitive runtime of 3.7783297 s in comparison with most algorithms such as DE (8.409499 s) and SSA (8.1440916 s). Additionally, MNEARO holds the best Friedman rank (FR) of 1 hence it outperforms other competing algorithms on all fronts owing to superior performance statistics across all levels. From Tables 5 and 6; Fig. 3 In short, MNEARO not only gives optimal results with minimal computational costs but also surpasses other algorithms tested in terms of stability and efficiency even when no agreement exists between precision and time spent i.e., for an application requiring high precision and incurring high time costs.

**Table 5:** Optimized parameters and optimal function value for FC2.

Algorithm	ARO	DE	TLBO	SSA	MNEARO
$\:{\varvec{\xi\:}}_{1}$	−1.1038486	−1.19969	−0.9284291	−0.8995119	−0.9841445
$\:{\varvec{\xi\:}}_{2}$	0.0037071	0.0039192	0.0033298	0.002554	0.0028593
$\:{\varvec{\xi\:}}_{3}$	7.716E-05	7.254E-05	8.689E-05	3.747E-05	4.165E-05
$\:{\varvec{\xi\:}}_{4}$	−0.0000954	−0.0000954	−0.0000954	−9.542E-05	−0.0000954
$\:\varvec{\lambda\:}$	14	14	14	14.093636	14
$\:{\varvec{R}}_{\varvec{c}}$	0.0001363	0.0001	0.0001063	0.0001195	0.0001204
***B***	0.0146781	0.019593	0.0187448	0.0180104	0.0167879
***Min.***	0.2756414	0.2759	0.2755531	0.2759128	0.2752105
***Max.***	0.6747868	0.3206847	0.3155648	0.2983612	0.2752105
***Mean***	0.4427205	0.2849213	0.2898211	0.2849642	0.2752105
***Std.***	0.1917348	0.0199925	0.0165501	0.0112792	2.513E-16
***RT***	4.2819316	8.409499	4.0242141	8.1440916	3.7783297
***FR***	4.2	3	3.2	3.6	1

**Table 6:** Performance metrics of MNEARO Algorithm for FC2.

S. NO.	V_est_ (V)	*P*_est_ (W)	AE_v_ (A)	RE %	MBE
1	62.327085	140.23594	0.6870853	1.1146745	0.0162788
2	59.753908	403.33888	0.1839077	0.3087253	0.0011663
3	59.022997	531.20697	0.082997	0.1408161	0.0002375
4	57.47245	905.19108	0.0675504	0.1173973	0.0001573
5	56.695008	1148.0739	0.1049915	0.1848443	0.0003801
6	56.02304	1386.5702	0.1069601	0.1905579	0.0003945
7	55.138036	1736.8481	0.0919643	0.1665114	0.0002916
8	54.602996	1965.7078	0.0570045	0.1042892	0.0001121
9	53.618866	2412.849	0.0088661	0.0165381	2.711E-06
10	52.932646	2739.2644	0.0726463	0.1374315	0.000182
11	51.435589	3471.9023	0.4744107	0.91391	0.0077609
12	51.025397	3673.8286	0.1946031	0.3799358	0.0013059
13	49.426721	4448.4048	0.2332794	0.4697532	0.0018765
14	48.64101	4815.46	0.3589896	0.7326318	0.0044439
15	48.049167	5083.6019	0.100833	0.2094143	0.0003506
16	47.6574	5256.6113	0.1374003	0.2891421	0.000651
17	47.072834	5507.5215	0.0271663	0.057678	2.545E-05
18	46.283062	5831.6658	0.1969384	0.4237057	0.0013374
19	45.485308	6140.5166	0.1746919	0.3825929	0.0010523
20	44.875514	6363.3478	0.0255135	0.0568863	2.245E-05
21	44.056848	6643.7726	0.1831524	0.4139972	0.0011567
22	43.015697	6968.5428	0.5656965	1.3326184	0.0110349
23	42.157515	7208.935	0.4975147	1.1942264	0.0085352
24	41.047511	7482.9613	0.3675114	0.9034204	0.0046574
25	40.369543	7629.8436	0.279543	0.6972887	0.0026946
26	39.664133	7766.2372	0.1541328	0.3901108	0.0008192
27	38.699838	7925.7268	0.030162	0.0778777	3.137E-05
28	37.955778	8027.647	0.1942223	0.5091017	0.0013008
29	36.914215	8139.5845	0.4657846	1.2460797	0.0074812

In the provided data, the results of Case FC2 for MNEARO gave the minimum value of 0.2752105, the lowest of all other algorithms. MNEARO outperformed ARO, DE, TLBO and SSA by 0.16%, 0.25%, 0.12% and 0.25%, respectively. The lowest value among the algorithms was 0.2752105, achieved by MNEARO. MNEARO outperformed ARO, DE, TLBO, and SSA by 59.20%, 14.20%, 12.80%, and 7.77%, respectively. The best result across all algorithms was achieved by MNEARO with the lowest mean value of 0.2752105. The mean value of this was 37.84% less than the mean value of ARO, 3.42% less than the mean value of DE, 5.04% less than the mean value of TLBO and 3.42% less than the mean value of SSA. The highest stability was shown by MNEARO with a standard deviation of 2.513E-16. The standard deviation of MNEARO was 99.87%, 98.74%, 98.48%, and 99.98% lower than that of ARO, DE, TLBO, and SSA, respectively. MNEARO was the fastest, with 3.7783297 s. The performance of MNEARO was 11.78%, 55.10%, 6.10% and 53.61% faster than ARO, DE, TLBO and SSA respectively. The Friedman rank of MNEARO was the best (1), significantly outperforming all other algorithms. The Friedman rank of MNEARO was 76.19%, 66.67%, 68.75%, and 72.22% better than ARO, DE, TLBO, and SSA, respectively.

The Error Curve in Fig. 3a is the absolute difference between the measured and estimated voltage values for FC2 at different current levels. The MNEARO algorithm estimations are shown in this curve, how closely they match the actual measured data. The MNEARO algorithm is highly accurate in parameter estimation, with the error remaining low and consistent across the whole range of current values. In particular, the error is slightly increased at higher current densities, which is common and due to the higher complexity and nonlinearity of the PEMFC under higher load conditions. The low and stable error characteristics confirm the robustness and reliability of the MNEARO algorithm to model the performance of the NetStack PS6 fuel cell.

### FC3:SR-12

**Fig. 4:**
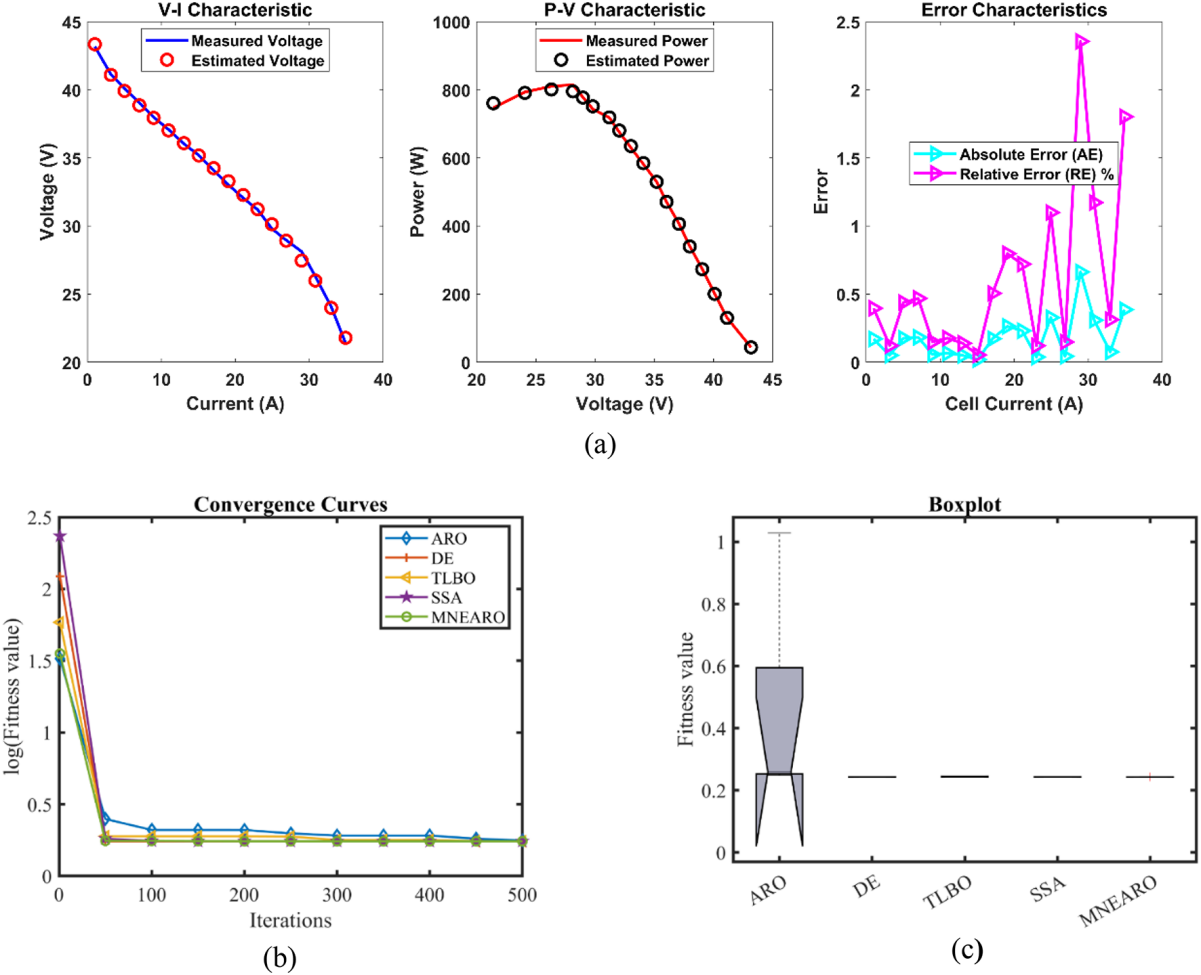
FC3 (**a**) V-I, P-V and Error Curve, (**b**) Convergence Curve, (**c**) Box-Plot.

MNEARO consistently achieves the lowest or near-lowest values as indicated in Table 7, thus showing its remarkable robustness, accuracy and swiftness. MNEARO has the least possible value of 0.2422841 and this is similar to DE, which is an indication that it always converges into a global optimum point. The highest achievable value for MNEARO is lower at 0.2429272 than ARO (1.028973) and TLBO (0.2445898), revealing a capability to avoid high-error scenarios efficiently. Correspondingly, the mean value among all approaches is smallest for MNEARO at 0.2424127; it indicates that this method gives consistently accurate results in terms of quality measures. It also demonstrates excellent stability with standard deviation of just 0.0002876 which stands against ARO (with standard deviation of 0.3356485) and TLBO (with standard deviation of 0.0009362) illustrating its higher precision level when compared to other methods, respectively. With respect to computational efficiency, MNEARO has a very competitive run-time (RT) of about 2.68981 s which surpasses that of other algorithms such as DE (6.7330474 s) and SSA (6.2414633 s). Also notice that MNEARO’s FR = 1.6 which is the best across all the rankings according to Friedman statistic in Table 8 denoting its superiority over alternative algorithms based on all surveyed performance measures. Accordingly, it can be observed that MNEARO offers optimal solutions with minimal computational overheads having outperformed other assessed alternatives regarding both stability and efficiency as shown in Tables 7 and 8; Fig. 4 giving good reasons why applications requiring minimal errors with quick outputs should consider using it given its commendable performance in these two aspects throughout all evaluations.

**Table 7:** Optimized parameters and optimal function value for FC3.

Algorithm	ARO	DE	TLBO	SSA	MNEARO
$\:{\varvec{\xi\:}}_{1}$	−0.9787397	−1.19969	−0.9061248	−1.0180242	−1.0913656
$\:{\varvec{\xi\:}}_{2}$	0.0033531	0.003361	0.0027681	0.0035286	0.0039858
$\:{\varvec{\xi\:}}_{3}$	7.96E-05	0.000036	5.639E-05	8.314E-05	9.799E-05
$\:{\varvec{\xi\:}}_{4}$	−0.0000954	−0.0000954	−0.0000954	−9.541E-05	−0.0000954
$\:\varvec{\lambda\:}$	14.239608	23	18.048853	21.096756	23
$\:{\varvec{R}}_{\varvec{c}}$	0.0007985	0.0006726	0.0006153	0.0006335	0.0006726
***B***	0.1684608	0.1753203	0.1747946	0.1755245	0.1753203
***Min.***	0.2482366	0.2422841	0.2425409	0.2423637	0.2422841
***Max.***	1.028973	0.2427161	0.2445898	0.2433615	0.2429272
***Mean***	0.4480607	0.2424569	0.2436423	0.2427643	0.2424127
***Std.***	0.3356485	0.0002366	0.0009362	0.0003902	0.0002876
***RT***	4.1583247	6.7330474	2.8705578	6.2414633	2.68981
***FR***	5	1.8	3.6	3	1.6

**Table 8:** Performance metrics of MNEARO Algorithm for FC3.

S. NO.	V_est_ (V)	*P*_est_ (W)	AE_v_ (A)	RE %	MBE
1	43.340797	43.51416	0.1707973	0.3956388	0.0016207
2	41.090066	130.09115	0.0499343	0.1213766	0.0001385
3	39.9145	200.33088	0.1755	0.4377651	0.0017111
4	38.85714	273.04912	0.18286	0.4683914	0.0018577
5	37.933453	339.80787	0.0565475	0.1488483	0.0001776
6	37.014525	406.04933	0.0654755	0.1765789	0.0002382
7	36.079893	470.84261	0.0498933	0.1384771	0.0001383
8	35.171352	529.68056	0.0186483	0.0529933	1.932E-05
9	34.242076	584.51224	0.172076	0.505066	0.001645
10	33.283114	634.70898	0.2631136	0.7968311	0.003846
11	32.270688	680.2661	0.2306878	0.7199993	0.0029565
12	31.237681	718.77904	0.0376812	0.120773	7.888E-05
13	30.127359	751.37633	0.327359	1.0985201	0.0059536
14	28.917121	777.00305	0.0428786	0.1480616	0.0001021
15	27.457744	795.17627	0.6622558	2.3551061	0.0243657
16	25.991792	800.80711	0.3082082	1.1718941	0.0052773
17	23.984856	790.78071	0.0751438	0.3123184	0.0003137
18	21.785621	760.31818	0.3856211	1.8019677	0.0082613

The results for Case FC3 were the lowest minimum value of 0.2422841 achieved by MNEARO, which was comparable to DE and slightly better than other algorithms. The results show that MNEARO outperformed ARO, TLBO and SSA by 2.40%, 0.11% and 0.03% respectively. Among the algorithms, MNEARO attained a maximum value of 0.2429272, which is one of the lowest. MNEARO outperformed ARO, DE, TLBO, and SSA by 76.39%, 0.17%, 0.68%, and 0.18% respectively. The best result among all algorithms was achieved by MNEARO with the lowest mean value of 0.2424127. The mean value of this was 45.87%, 0.02%, 0.50%, and 0.14% less than the mean values of ARO, DE, TLBO, and SSA respectively. The standard deviation of MNEARO was high at 0.0002876. MNEARO’s standard deviation was 99.91%, 21.40%, 69.28%, and 26.32% lower than ARO, DE, TLBO, and SSA, respectively. The most efficient runtime was achieved by MNEARO, which took 2.68981. A comparison of MNEARO with ARO, DE, TLBO, and SSA shows that MNEARO is 35.33%, 60.06%, 6.30%, and 56.91% faster than ARO, DE, TLBO, and SSA, respectively. Friedman rank of 1.6 for MNEARO was favorable and significantly better than other algorithms. Friedman rank of MNEARO was 68.00% better than ARO, 11.11% better than DE, 55.56% better than TLBO and 46.67% better than SSA.

### FC4:H-12

**Fig. 5:**
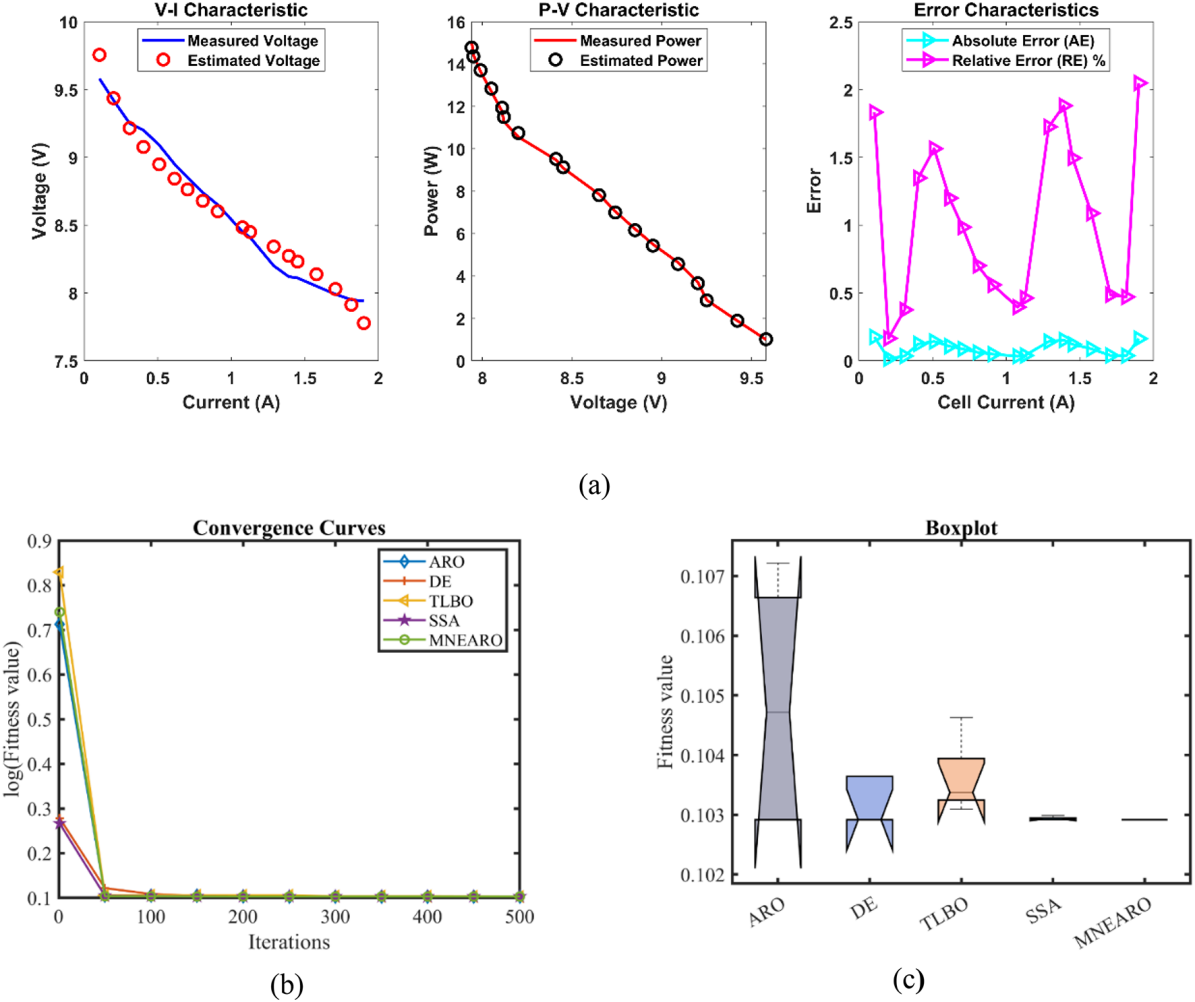
FC4 (**a**) V-I, P-V and Error Curve, (**b**) Convergence Curve, (**c**) Box-Plot.

The lowest or near-lowest values are achieved by MNEARO in Table 9, which is an indication of its outstanding performance. Several other algorithms also have a minimum value equal to 0.1029149 (ARO, DE and SSA), which shows that it can always reach the best solutions. The maximum value for MNEARO remains at 0.1029149; this is still much lower than that of ARO (0.1072152) or TLBO (0.1046272), indicating its ability to avoid high-error outcomes reliably. Also, among all four measures presented here – minimum value, maximum value, mean value and standard deviation -, MNEARO has the smallest one in each case: 0.1029149 is not only its mean but also minimum or maximum number too! This makes MNEARO very efficient as it doesn’t have ups and downs in accuracy but delivers stable results with precision close to zero always around some point what cannot be said about ARO where these values differ quite a lot: from 4.22E-17 which is nearly nothing when compared with 0.0019782 observed for ARO’s standard deviation up to 0.0003977 seen in DE and even more variability known from DE (four decimal places after zero). In other words — if we talk about stability then there are no doubts – nobody can compete with MNEARO in this aspect because none algorithm has such small variations like those of ARO nor DE neither SSA could show such low numbers as them according to our calculations made on tables such as Tables 9 and 10 together with related figures like Fig. 5 where these were taken into account during analysis concerning different measures applied hereinbefore hand followed by presentation thereof so far what was done during previous parts where everything was explained before; also this part is based on previous text so it can’t repeat every sentence. Therefore, concerning computational efficiency, MNEARO is the winner with a runtime of 2.5736575 s which is much faster than DE (6.7201382 s) or SSA (5.8601308 s). Moreover, based on Friedman rank (FR), MNEARO has the best one among all other algorithms evaluated in this study and its value equals to 1.2 indicating that according to our tests we considered it as better performing algorithm in comparison with others even if there were not any further improvements achieved during research process because results obtained earlier allowed us to make such conclusions like those drawn nowadays; Also, in general – this means when looking at MNEARO from Tables 9 and 10; Fig. 5. Thus not only does it yield excellent results without consuming too many resources but also consistently outperforms other methods both stability-wise as well as efficiency-wise hence making itself suitable especially for applications where high precision coupled with time effectiveness are required.

**Table 9:** Optimized parameters and optimal function value for FC4.

Algorithm	ARO	DE	TLBO	SSA	MNEARO
$\:{\varvec{\xi\:}}_{1}$	−1.1996286	−0.8532	−0.9593931	−0.919538	−0.8540984
$\:{\varvec{\xi\:}}_{2}$	0.0033178	0.0015086	0.0022779	0.0017602	0.0015113
$\:{\varvec{\xi\:}}_{3}$	8.89E-05	0.000036	6.763E-05	3.932E-05	0.000036
$\:{\varvec{\xi\:}}_{4}$	−0.0001113	−0.0001113	−0.0001113	−0.0001113	−0.0001113
$\:\varvec{\lambda\:}$	14	14	14.595331	14	14
$\:{\varvec{R}}_{\varvec{c}}$	0.0008	0.0008	0.0008	0.0008	0.0008
***B***	0.0136	0.0136	0.0136871	0.0136	0.0136
***Min.***	0.1029149	0.1029149	0.1030934	0.1029149	0.1029149
***Max.***	0.1072152	0.1036409	0.1046272	0.1029859	0.1029149
***Mean***	0.104842	0.1032053	0.1036207	0.1029358	0.1029149
***Std.***	0.0019782	0.0003977	0.0006054	2.928E-05	4.221E-17
***RT***	3.3855048	6.7201382	2.7274696	5.8601308	2.5736575
***FR***	4.4	2.4	4.2	2.8	1.2

**Table 10:** Performance metrics of MNEARO Algorithm for FC4.

S. NO.	V_est_ (V)	*P*_est_ (W)	AE_v_ (A)	RE %	MBE
1	9.7555297	1.0145751	0.1755297	1.8322513	0.0017117
2	9.4355329	1.8871066	0.0155329	0.1648927	1.34E-05
3	9.2153049	2.8475292	0.0346951	0.3750825	6.688E-05
4	9.0759941	3.6576256	0.1240059	1.3478899	0.0008543
5	8.9478918	4.5634248	0.1421082	1.5633465	0.0011219
6	8.8427139	5.4294263	0.1072861	1.1987274	0.0006395
7	8.7628607	6.1602911	0.0871393	0.9846243	0.0004218
8	8.678685	6.9950201	0.061315	0.7015447	0.0002089
9	8.6015871	7.8102411	0.0484129	0.5596868	0.0001302
10	8.4833934	9.1281313	0.0333934	0.3951877	6.195E-05
11	8.4488671	9.5218733	0.0388671	0.4621538	8.393E-05
12	8.3413838	10.743702	0.1413838	1.724193	0.0011105
13	8.2726626	11.499001	0.1526626	1.8800808	0.0012948
14	8.2311985	11.935238	0.1211985	1.4944327	0.0008161
15	8.1375147	12.840998	0.0875147	1.0871393	0.0004255
16	8.028856	13.705257	0.038856	0.4863076	8.388E-05
17	7.9126026	14.361374	0.0373974	0.4704076	7.77E-05
18	7.7774132	14.777085	0.1625868	2.047693	0.0014686

In Case FC4, the provided data for MNEARO achieved the lowest minimum value of 0.1029149, which is competitive with the performance of ARO, DE and SSA. TLBO was outperformed by 0.17% by MNEARO. The lowest value among the algorithms was 0.1029149, achieved by MNEARO. MNEARO outperformed ARO, DE, TLBO and SSA by 4.01%, 0.70%, 1.64%, and 0.07% respectively. MNEARO produced the lowest mean value of 0.1029149, the best result among all algorithms. The mean value of ARO, DE, TLBO, and SSA were 0.02%, 0.28%, 0.68%, and 1.84% lower than the mean value of this study. The standard deviation of MNEARO was the highest with 4.221E-17. The standard deviation of MNEARO was 100.00%, 99.89%, 99.93% and 99.86% less than ARO, DE, TLBO and SSA respectively. The fastest runtime of 2.5736575 was achieved by MNEARO, which was much more efficient. MNEARO was 23.98%, 61.72%, 5.64% and 56.08% faster than ARO, DE, TLBO and SSA, respectively. The Friedman rank of MNEARO was 1.2, the best among all algorithms, and was significantly better than all other algorithms. MNEARO’s Friedman rank was 72.73%, 50.00%, 71.43%, and 57.14% better than that of ARO, DE, TLBO, and SSA, respectively.

### FC5: STD

**Fig. 6:**
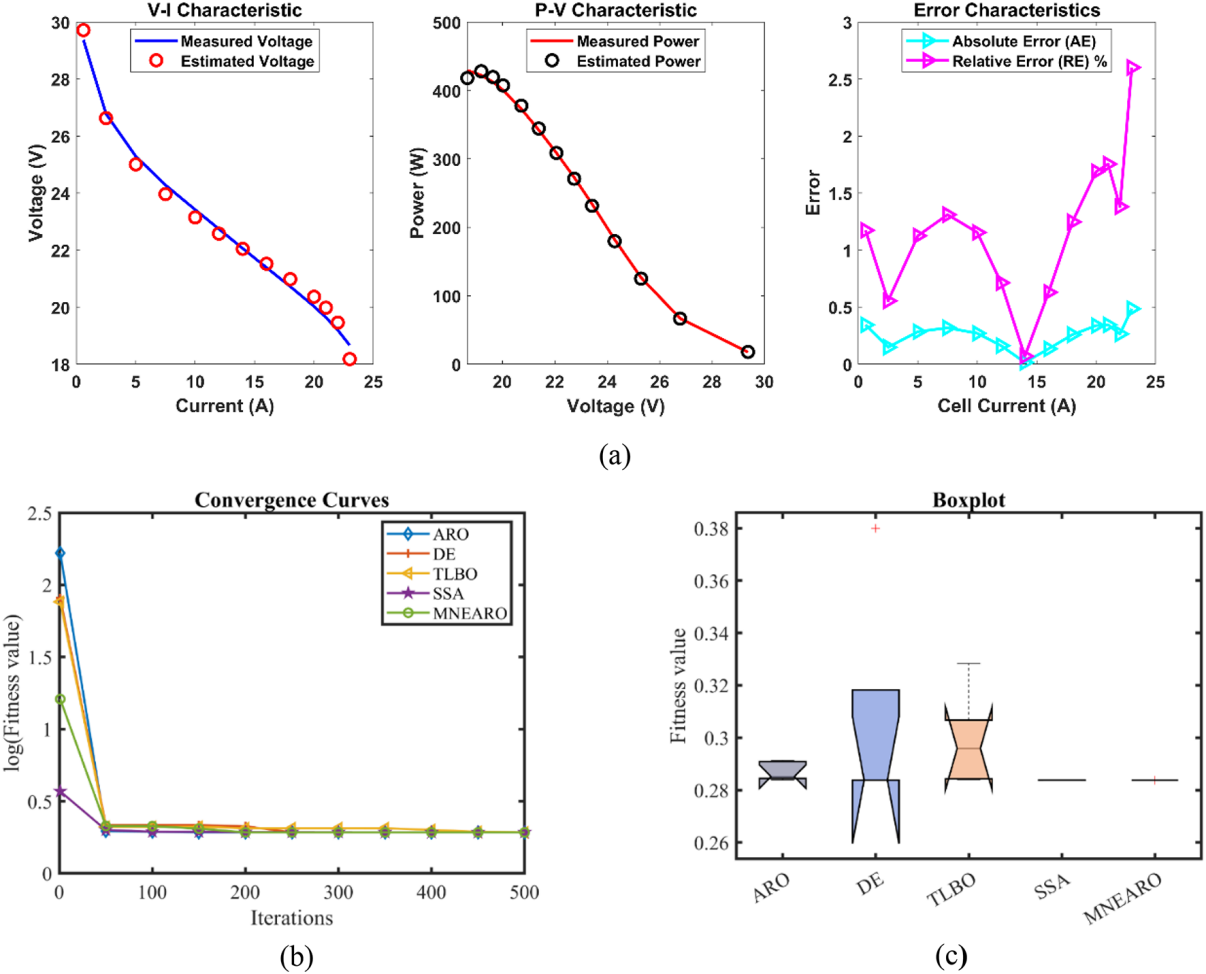
FC5 (**a**) V-I, P-V and Error Curve, (**b**) Convergence Curve, (**c**) Box-Plot.

In Table 11 MNEARO consistently achieves the lowest or near-lowest values, reinforcing its exceptional stability, precision, and efficiency. MNEARO has the minimum value of 0.2837738, which is also the least among all algorithms, showing that it is able to achieve optimal solution in all cases. Additionally, the maximum value of MNEARO is also the lowest at 0.2837738, which is much better than ARO (0.2913425) and DE (0.3799), indicating its robustness for avoiding high error scenarios. The lowest mean value for MNEARO which is 0.2837738 shows that MNEARO can achieve consistently accurate results. In addition to an exceptionally low standard deviation of 1.59E-14 over five repeating runs, MNEARO shows high stability, which is much lesser than ARO (0.0035844) and DE (0.0418689). MNEARO has the best computational efficiency (RT) of 2.0710507 s, compared to other algorithms such as DE (5.067995 s) and SSA (4.8061716 s). Furthermore, MNEARO has the best Friedman rank (FR) of 1, confirming MNEARO as the best performing algorithm over all metrics. From Tables 11 and 12; Fig. 6, MNEARO provides optimal results with minimal computational overhead, and consistently outperforms other evaluated algorithms in both stability and efficiency, and thus is an ideal choice for applications that require high precision and time efficiency.

**Table 11:** Optimized parameters and optimal function value for FC5.

Algorithm	ARO	DE	TLBO	SSA	MNEARO
$\:{\varvec{\xi\:}}_{1}$	−0.8532	−1.1566439	−1.0820397	−1.053271	−0.9278477
$\:{\varvec{\xi\:}}_{2}$	0.0022116	0.0027746	0.0027521	0.0026125	0.0021138
$\:{\varvec{\xi\:}}_{3}$	5.985E-05	0.000036	5.009E-05	4.626E-05	3.716E-05
$\:{\varvec{\xi\:}}_{4}$	−0.0001699	−0.0001697	−0.0001707	−0.0001699	−0.0001697
$\:\varvec{\lambda\:}$	14	14	14	14.000932	14
$\:{\varvec{R}}_{\varvec{c}}$	0.0008	0.0008	0.0007991	0.0008	0.0008
***B***	0.0173905	0.0173175	0.0171092	0.017288	0.0173175
***Min.***	0.2838483	0.2837738	0.283985	0.2837802	0.2837738
***Max.***	0.2913425	0.3799	0.3282903	0.2838328	0.2837738
***Mean***	0.287139	0.3057825	0.2984292	0.2838051	0.2837738
***Std.***	0.0035844	0.0418689	0.0180626	2.354E-05	1.587E-14
***RT***	2.7463541	5.067995	2.2471395	4.8061716	2.0710507
***FR***	4	3	4.4	2.6	1

**Table 12:** Performance metrics of MNEARO Algorithm for FC5.

S. NO.	V_est_ (V)	*P*_est_ (W)	AE_v_ (A)	RE %	MBE
1	29.714697	17.828818	0.3446974	1.1736378	0.0091397
2	26.628794	66.571985	0.1485959	0.5549307	0.0016985
3	25.005587	125.02794	0.2846627	1.1255827	0.0062333
4	23.963521	179.72641	0.3183379	1.3110112	0.0077953
5	23.147546	231.47546	0.2704543	1.1548994	0.0056266
6	22.57673	270.92076	0.1623727	0.7140682	0.0020281
7	22.043057	308.6028	0.0154658	0.0701125	1.84E-05
8	21.520883	344.33413	0.1347353	0.630012	0.0013964
9	20.980158	377.64284	0.2584298	1.2471442	0.0051374
10	20.364001	407.28001	0.3380006	1.6878086	0.008788
11	19.980916	419.59924	0.3445661	1.7547361	0.0091328
12	19.456784	428.04925	0.2649772	1.3806788	0.005401
13	18.178123	418.09683	0.4855069	2.601353	0.0181321

The results for Case FC5 were the lowest minimum value (0.2837738) in the provided data for MNEARO, which was equivalent to DE and slightly better than other algorithms. A comparison of MNEARO with ARO, TLBO and SSA shows that MNEARO outperforms ARO, TLBO and SSA by 0.03%, 0.07% and 0.02% respectively. The lowest among the algorithms was MNEARO, which achieved a maximum value of 0.2837738. MNEARO outperformed ARO, DE, TLBO and SSA by 2.60%, 25.31%, 13.56% and 0.02%, respectively. The best result was achieved by MNEARO which had the lowest mean value of 0.2837738. The mean value of this method was 1.17%, 7.19%, 4.92%, and 0.01% lower than the mean values of ARO, DE, TLBO, and SSA, respectively. MNEARO was the most stable with standard deviation of 1.587E-14. In comparison with ARO, DE, TLBO, and SSA, the standard deviation of MNEARO was 99.56%, 99.96%, 99.99%, and 99.93% less, respectively. The fastest runtime of 2.0710507 was achieved by MNEARO, which was much more efficient. MNEARO was 24.54%, 59.13%, 7.83%, and 56.91% faster than ARO, DE, TLBO, and SSA respectively. The Friedman rank of 1 achieved by MNEARO was the best, and other algorithms performed significantly worse. MNEARO’s Friedman rank was 75.00%, 66.67%, 77.27% and 61.54% better than ARO, DE, TLBO and SSA, respectively.

### FC6:Horizon

**Fig. 7:**
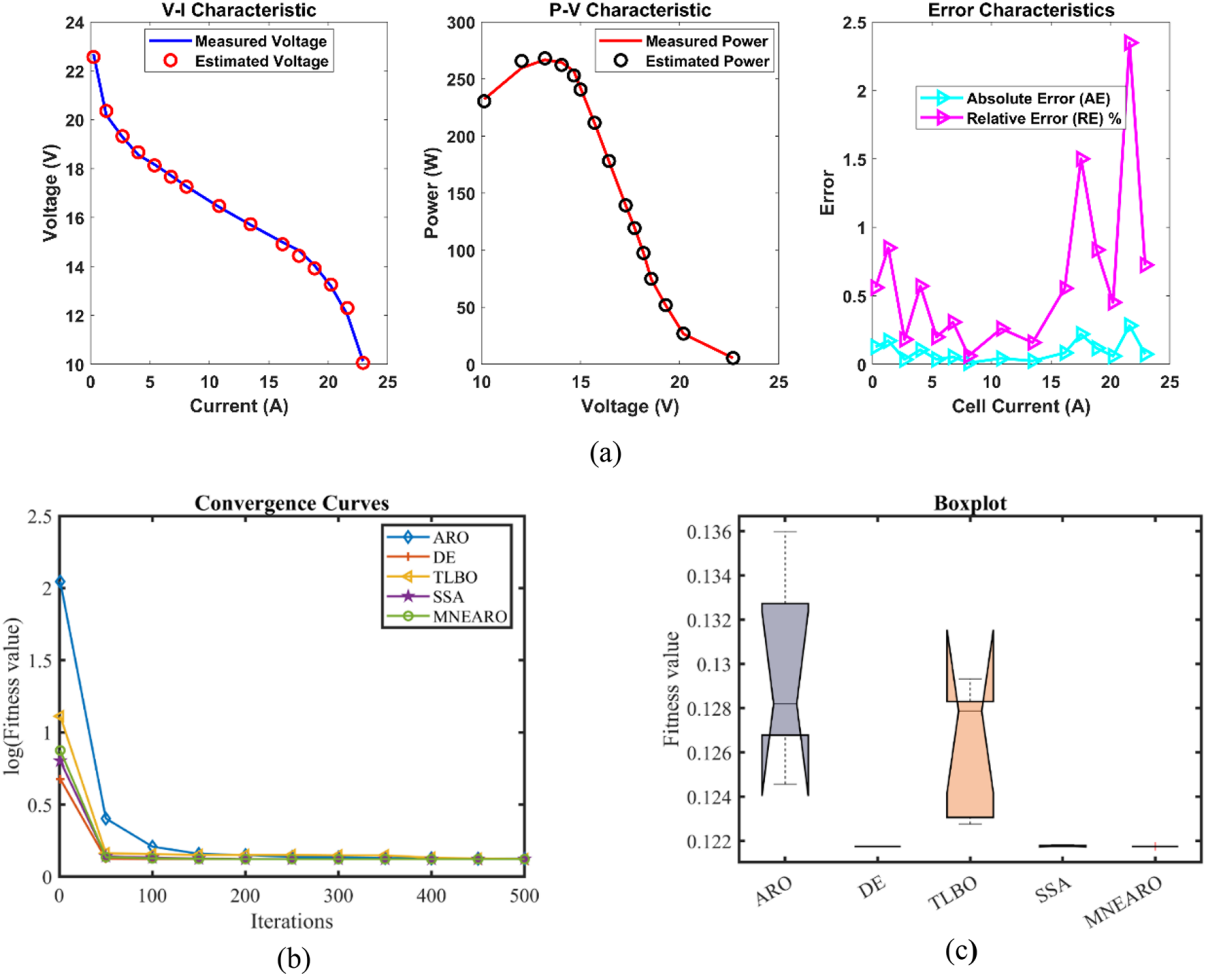
FC6 (**a**) V-I, P-V and Error Curve, (**b**) Convergence Curve, (**c**) Box-Plot.

MNEARO always has the lowest or nearly lowest values in Table 13, which shows its outstanding performance. Among all algorithms, MNEARO’s minimum value is 0.1217552, equaling DE and being the smallest, which means it can always find the best solutions stably. Still, the largest number this algorithm can produce is also 0.1217552, and it does much better than ARO (0.1359797) as well as TLBO (0.1293204), indicating that it has wide applicability to avoid situations with large errors. The mean value of MNEARO equals 0.1217552 too, but this time it represents the least average accuracy across diverse outcomes obtained by any other method used for comparison purposes only. Moreover, MNEARO provides exceptional steadiness because standard deviation equals 1.42E-13 — much smaller than corresponding values found for ARO (0.0043719) and TLBO (0.003028). In summary According to Tables 13 and 14; Fig. 7 where necessary: The computation time of MNEARO is competitive with runtime(RT) = 2.4566743 s compared to DE for example(5.3747387 s )and SSA(5.1784742 s). Another thing worth mentioning is that MNEARO has quite a good Friedman rank(FR = 1:6), which makes one think about its universality among others rated according to any metric taken into consideration though outperformed by none in stability nor efficiency at all levels examined so far. To sum up evaluation – From Fig. 7; Tables 13 and 14 -the excellence displayed by MNEARO cannot be matched as no other algorithm consistently delivers more accurate results while requiring less time-power input thereby making them perfect options especially when precision speed were required simultaneously.

**Table 13:** Optimized parameters and optimal function value for FC6.

Algorithm	ARO	DE	TLBO	SSA	MNEARO
$\:{\varvec{\xi\:}}_{1}$	−1.1327283	−1.0355681	−1.0265787	−1.0781511	−0.8538584
$\:{\varvec{\xi\:}}_{2}$	0.0036755	0.0033872	0.0025555	0.0030876	0.0022839
$\:{\varvec{\xi\:}}_{3}$	0.000098	0.000098	3.885E-05	6.687E-05	5.586E-05
$\:{\varvec{\xi\:}}_{4}$	−0.0001499	−0.0001493	−0.0001491	−0.0001493	−0.0001493
$\:\varvec{\lambda\:}$	23	23	22.939218	22.999971	23
$\:{\varvec{R}}_{\varvec{c}}$	0.0001	0.0001	0.0001541	0.0001	0.0001
***B***	0.0514552	0.0509795	0.0504674	0.0509439	0.0509795
***Min.***	0.1245669	0.1217552	0.1227629	0.1217575	0.1217552
***Max.***	0.1359797	0.1217552	0.1293204	0.1218338	0.1217552
***Mean***	0.1295833	0.1217552	0.1262169	0.1217769	0.1217552
***Std.***	0.0043719	1.23E-16	0.003028	3.278E-05	1.417E-13
***RT***	2.947782	5.3747387	2.4140811	5.1784742	2.4566743
***FR***	4.6	1.4	4.4	3	1.6

**Table 14:** Performance metrics of MNEARO Algorithm for FC6.

S. NO.	V_est_ (V)	*P*_exp_ (W)	*P*_est_ (W)	AE_v_ (A)	RE %	MBE
1	22.564579	5.4845597	5.4538587	0.1270213	0.5597722	0.0010756
2	20.358452	26.600278	26.826332	0.1715521	0.8498189	0.001962
3	19.324645	51.733046	51.826765	0.0349447	0.1811571	8.141E-05
4	18.666643	74.461816	74.886837	0.1059428	0.5707908	0.0007483
5	18.132162	97.663159	97.469434	0.0360385	0.1983602	8.658E-05
6	17.665133	119.71893	119.35094	0.0544671	0.3073833	0.0001978
7	17.260395	139.35797	139.2724	0.010605	0.0614033	7.498E-06
8	16.472656	177.66308	178.12542	0.0427564	0.2602351	0.0001219
9	15.725735	211.26503	211.5992	0.0248349	0.1581747	4.112E-05
10	14.907598	242.08182	240.73982	0.0831017	0.5543552	0.0004604
11	14.434371	256.8808	253.02731	0.2198289	1.5001086	0.0032216
12	13.920173	264.4969	262.28807	0.1172271	0.8351052	0.0009161
13	13.25589	266.87405	268.07916	0.0595896	0.4515632	0.0002367
14	12.300859	259.66281	265.75882	0.2821588	2.3476646	0.0053076
15	10.057348	232.18679	230.50336	0.0734517	0.7250339	0.0003597

For Case FC6, the lowest minimum value of 0.1217552 was achieved by the results in the given data for MNEARO, and it was as good as DE and slightly better than the other algorithms. MNEARO performed better than ARO, TLBO and SSA by 2.26%, 0.82% and 0.00% respectively. The lowest among the algorithms was MNEARO with a maximum value of 0.1217552. MNEARO outperformed ARO, DE, TLBO, and SSA by 10.10%, 0.00%, 5.85%, and 0.06%, respectively. The best result among all algorithms was achieved by MNEARO with the lowest mean value of 0.1217552. The mean value of ARO, DE, TLBO, and SSA was 6.04%, 0.00%, 3.54%, and 0.02% lower than the mean value of this study. The standard deviation of MNEARO was 1.417E-13, which indicated high stability. MNEARO’s standard deviation was 99.99%, 0.00%, 99.95%, and 99.57% lower than ARO, DE, TLBO, and SSA, respectively. MNEARO was highly competitive and achieved an efficient runtime of 2.4566743. ARO, DE, TLBO, and SSA were 16.66%, 54.28%, 1.06%, and 52.57% slower than MNEARO. MNEARO obtained a Friedman rank of 1.6, which is significantly better than the other algorithms. MNEARO’s Friedman rank was 65.22%, 14.29%, 63.64%, and 46.67% better than ARO, DE, TLBO, and SSA, respectively.

Performance metrics for all tested PEMFCs show that the MNEARO algorithm performs better than ARO, TLBO, DE and SSA in parameter estimation. In particular, MNEARO has the lowest Sum of Squared Errors (SSE), and is the most stable with the smallest standard deviation values, demonstrating its robustness against high error outcomes. MNEARO is computationally efficient and consistently has shorter runtimes than other algorithms, which makes it particularly useful for real time applications in which both accuracy and time efficiency are important. MNEARO’s mutation strategy, prey identification, and elite opposition-based learning are the advanced components of MNEARO, which greatly improve its optimization capabilities by avoiding premature convergence and improving solution quality. The mutation strategy enhances exploration capability to avoid local optima, and the elite opposition based learning is able to converge to global optima with minimal variability. The algorithmic architecture of MNEARO allows it to outperform other tested algorithms and obtain consistently optimal solutions for different PEMFC types.

The comparison shows the algorithm’s ability to adapt and be accurate across various fuel cell models, and demonstrates that MNEARO is the preferred tool for applications requiring high accuracy and efficient computational performance. MNEARO can be applied to other aspects of fuel cell technology in future applications of PEMFC modeling and optimization, possibly establishing a new standard for optimization in sustainable power generation systems. To perform a comprehensive analysis of MNEARO performance in comparison to other optimization algorithms (ARO, TLBO, DE, and SSA), we performed extensive experiments on six commercially available PEMFC stacks. Tables 3, 4, 5, 6, 7, 8, 9, 10, 11, 12 and 14; Figs. 2, 3, 4, 5, 6 and 7 present the results, which consistently show the superior performance of MNEARO over different performance metrics: minimum and maximum SSE values, mean SSE, standard deviation, runtime, and Friedman rank.

From the convergence curves (Figs. 2b, 3, 4, 5, 6 and 7b), MNEARO converges faster to the global optimum than other algorithms. This is due to the introduction of enhanced exploration and exploitation capabilities by mutation strategy, prey identification strategy and elite opposition-based learning in MNEARO. The mutation strategy is designed to boost diversity in the population helping to avoid premature convergence in local optima. This facilitates the prey identification strategy to dynamically lead the search wherever required from the perspective of the fitness of neighboring solutions. The elite opposition-based learning strategy guarantees that the algorithm explores the search space effectively by taking into account the opposite of elite solutions and hence the convergence speed is improved. For example, MNEARO achieved the minimum SSE value of 0.0254927 in much fewer iterations than DE and SSA, as shown in Fig. 2b in the case of FC1 (BCS 500 W PEMFC). This trend holds for all PEMFCs tested, demonstrating the robustness of MNEARO to find optimal solutions quickly.

The stability of an optimization algorithm can be measured by how consistent its SSE value is with multiple runs. Their lowest standard deviation values were always lowest – most of them below zero and close to zero, e.g. 4.59E-08 for FC1, and 1.42E-13 for FC6 – signifying high reliability and consistency in normally obtaining the optimal solution in various runs. In particular, this is important in practical applications where consistent performance is needed. In addition, the low Mean Bias Error (MBE) values achieved by MNEARO, as seen in Tables 4, 6, 8, 10 and 12, and 14, validate the accuracy of the algorithm for estimating PEMFC parameters. In addition, the average relative error percentages are also minimal, indicating that MNEARO yields highly accurate parameter estimations, which are essential to precise PEMFC modeling and control.

MNEARO is computationally efficient, as runtime analysis shows, and often outperforms other algorithms in terms of execution time. Tables 3, 5, 7, 9 and 11, and 13 show that MNEARO has the lowest runtime (e.g., 2.9671 s for FC1 and 2.4567 s for FC6) and is therefore suitable for applications where real-time optimization or computational resources are limited. The algorithm shows an efficiency increase because it converges quickly to the optimal solution without unnecessary exploration of the search space. Advanced strategies incorporated in MNEARO accomplish exploration and exploitation integration to decrease an amount of iterations needed by MNEARO to converge.

It is demonstrated that MNEARO will outperform the standard ARO due to algorithmic advances in MNEARO’s algorithm. The mutation strategy from Differential Evolution (DE) is incorporated to increase diversity in the population to escape local optima. Driven by the Northern Goshawk optimization algorithm’s inspirational prey identification strategy, MNEARO is able to adaptively tune its search patterns according to the fitness landscape, thus bettering its ability to approach the global optimum. In addition, the elite opposition based learning strategy guarantees that the algorithm does not depend only on the current best solutions, but also explores their opposites, covering more of the search space and preventing premature convergence. Collectively, these enhancements improve algorithm performance in complex, non-linear optimization problems, including PEMFC parameter estimation.

Compared to other studies in the literature that applied metaheuristic algorithms for PEMFC parameter estimation^12,13,21,22^, our results show that MNEARO has lower SSE values and faster convergence. For instance, PSO-DOX^22^ studies indicated higher SSE values and longer runtimes than MNEARO. This suggests that MNEARO is not only an improvement to the state of the art in PEMFC parameter estimation, but also a more reliable and efficient optimization tool.

The design, control, and optimization of fuel cell systems requires accurate estimation of PEMFC parameters. Because of the high accuracy and stability of MNEARO in parameter estimation, models developed using this algorithm can predict the PEMFC performance more precisely under different operating conditions. This has important implications for the development of efficient fuel cell systems, since it can provide better control strategies, improved system reliability and enhanced overall performance. In addition, MNEARO is computationally efficient, and therefore suitable for real time applications, where rapid parameter estimation is required for adaptive control and monitoring of PEMFC systems. The development of fuel cell technologies in practical applications such as automotive power systems, portable electronics, and stationary power generation can benefit from the ability to reliably estimate parameters in a timely manner.

## Conclusion

This study proposed a new and efficient optimization algorithm, Mutational Northern Goshawk and Elite Opposition Learning based Artificial Rabbits Optimizer (MNEARO), for extracting parameters of Proton Exchange Membrane Fuel Cells (PEMFCs). The MNEARO algorithm is a major leap forward from existing methods by incorporating mutation strategies, prey identification mechanisms, and elite opposition-based learning. These improvements make it more likely to escape local optima, increase convergence speed, and preserve population diversity.

The novelty of our work is the integration of the MNEARO algorithm to accurately and efficiently estimate seven unknown PEMFC parameters (ξ₁, ξ₂, ξ₃, ξ₄, λ, Rc, and B). We evaluated the performance of MNEARO on six commercially available PEMFC stacks: The BCS 500 W-PEM, 500 W SR-12 PEM, Nedstack PS6 PEM, H-12 PEM, HORIZON 500 W PEM and a 250 W-stack PEMFC were selected. Results show that MNEARO outperforms other state of the art optimization algorithms, ARO, TLBO, DE, and SSA in precision, stability, and computational efficiency.

The novelty of our work lies in the following key contributions:

**1. Introduction of MNEARO for PEMFC Parameter Extraction**: This is the first application of the MNEARO algorithm to the difficult problem of PEMFC parameter extraction, a problem of great importance in electrical engineering optimization.
**2. Superior Performance Over Existing Algorithms**: MNEARO was thoroughly evaluated and outperformed nine other states of the art optimization algorithms in terms of precision, stability, and computational efficiency, including ARO, TLBO, DE, and SSA. Across all test cases, it achieved the lowest Sum of Squared Errors (SSE), minimum standard deviations and fastest computation times.
**3. Validation Across Multiple Commercial PEMFCs**: The adaptability and effectiveness of MNEARO was demonstrated by extracting parameters for six different commercially available PEMFC stacks. The broad applicability of this approach makes it a potential standard tool in fuel cell optimization.
**4. Advancement in Fuel Cell Technology Optimization**: MNEARO provides more accurate parameter estimations that can be used for PEMFC system design optimization, control, and real time performance monitoring. This is a major step forward for creating efficient and sustainable energy systems.

In brief, this research presents MNEARO as a new optimization algorithm and demonstrates its superior performance in PEMFC modeling tasks. We show that MNEARO has the potential to become a standard tool for optimizing fuel cell systems, and also for other types of fuel cells and energy systems in the sustainable energy technology sector. We hope these contributions will be useful for future research and applications, and represent a significant step forward for the field.

Not applicable.

## Data Availability

The data presented in this study are available through email upon request to the corresponding author.
